# Daple is a novel non-receptor GEF required for trimeric G protein activation in Wnt signaling

**DOI:** 10.7554/eLife.07091

**Published:** 2015-06-30

**Authors:** Nicolas Aznar, Krishna K Midde, Ying Dunkel, Inmaculada Lopez-Sanchez, Yelena Pavlova, Arthur Marivin, Jorge Barbazán, Fiona Murray, Ulrich Nitsche, Klaus-Peter Janssen, Karl Willert, Ajay Goel, Miguel Abal, Mikel Garcia-Marcos, Pradipta Ghosh

**Affiliations:** 1 Department of Medicine, University of California, San Diego, San Diego, United States; 2 Department of Biochemistry, Boston University School of Medicine, Boston, United States; 3 Translational Medical Oncology Laboratory, Health Research Institute of Santiago, Servizo Galego de Saúde, Santiago de Compostela, Spain; 4 Department of Surgery, Klinikum rechts der Isar, Technische Universität München, Munich, Germany; 5 Sanford Consortium for Regenerative Medicine, University of California, San Diego, La Jolla, California, United States; 6 Division of Gastroenterology, Department of Internal Medicine and Charles A Sammons Cancer Center and Baylor Research Institute, Baylor University Medical Center, Dallas, Texas, United States; 7 Moores Cancer Center, University of California, San Diego, San Diego, United States; Howard Hughes Medical Institute, Johns Hopkins University School of Medicine, United States

**Keywords:** G proteins, GEF, PI3K/Akt, tumor suppressor, Rac1, human

## Abstract

Wnt signaling is essential for tissue homeostasis and its dysregulation causes cancer. Wnt ligands trigger signaling by activating Frizzled receptors (FZDRs), which belong to the G-protein coupled receptor superfamily. However, the mechanisms of G protein activation in Wnt signaling remain controversial. In this study, we demonstrate that FZDRs activate G proteins and trigger non-canonical Wnt signaling via the Dishevelled-binding protein, Daple. Daple contains a Gα-binding and activating (GBA) motif, which activates Gαi proteins and an adjacent domain that directly binds FZDRs, thereby linking Wnt stimulation to G protein activation. This triggers non-canonical Wnt responses, that is, suppresses the β-catenin/TCF/LEF pathway and tumorigenesis, but enhances PI3K-Akt and Rac1 signals and tumor cell invasiveness. In colorectal cancers, Daple is suppressed during adenoma-to-carcinoma transformation and expressed later in metastasized tumor cells. Thus, Daple activates Gαi and enhances non-canonical Wnt signaling by FZDRs, and its dysregulation can impact both tumor initiation and progression to metastasis.

**DOI:** http://dx.doi.org/10.7554/eLife.07091.001

## eLife digest

Our cells need to be able to communicate with each other to coordinate many processes in the body, including the formation and maintenance of our organs. A system called Wnt signaling allows cells in different tissues to communicate. During Wnt signaling, one cell releases a protein called Wnt that then binds to a receptor protein known as Frizzled on the surface of another cell. This triggers a cascade of signaling events in the second cell, which leads to changes in the activity of particular genes. Wnt signaling is vital to many processes in cells, and any defects can cause cancer and other severe diseases.

Frizzled is a member of a large family of receptor proteins known as the G protein-coupled receptors (or GPCRs for short). These proteins can bind to other proteins called G proteins. When a GPCR is active, it can activate the G protein, which can then interact with several other signal proteins to amplify the signal from the GPCR.

However, there is currently no firm evidence that Frizzled can directly bind to G proteins. Some researchers have suggested that it may interact with G proteins via another ‘linker’ protein, but no such protein has yet been identified. Here, Aznar et al. investigated how Frizzled can activate G proteins in human cells. The experiments revealed that a protein called Daple can bind to both Frizzled and the G proteins when the cells are exposed to Wnt to activate the G proteins.

Aznar et al. show that Daple can act as a ‘tumor suppressor’ that reduces the risk of healthy cells becoming cancerous and can inhibit the growth of tumors. However, the amount of Daple increases in some tumor cells in the later stages of cancer, which makes it easier for these cells to spread around the body. Aznar et al. also observed that Daple was present at different levels in the late-stage tumor cells taken from a variety of cancer patients. Patients with higher levels of Daple were less likely to have a positive outcome from their cancer treatment, and their illness progressed more rapidly than patients with lower levels of Daple.

The next challenge is to understand what causes Daple to switch from its tumor suppressor role to one that promotes the spread of tumors in the later stages of disease.

**DOI:** http://dx.doi.org/10.7554/eLife.07091.002

## Introduction

The Wnt signaling pathway plays a crucial role in embryonic development, in tissue regeneration, and in many other cellular processes including cell fate, adhesion, polarity, migration, and proliferation. Dysregulated expression of components within the Wnt pathway triggers many diseases, and most importantly, heralds cancer (Klaus and Birchmeier, 2008).

Of the multiple known Wnt proteins, some preferentially trigger the well-characterized canonical pathway, which enhances the stability, nuclear localization and activity of β-catenin, and the downstream activation of genes targeted by the TCF/LEF transcription machinery. Other Wnts, for example, Wnt5a deviate from this canonical paradigm, and trigger so-called non-canonical pathways (Kühl et al., 2000; Niehrs, 2001; Winklbauer et al., 2001). Among other events, these non-canonical pathways induce the elevation of intracellular Ca^2+^ and activation of the small G proteins RhoA and Rac1, which regulate polarized cell movements and the planar polarity of epithelial cells (Sheldahl et al., 1999; Kühl et al., 2000; Mayor and Theveneau, 2014). Of critical importance, non-canonical Wnt signaling antagonizes the canonical Wnt pathway (Torres et al., 1996; Olson and Gibo, 1998; Ishitani et al., 2003), although it is unclear how this occurs. Despite the lack of molecular mechanisms, dysregulation of the non-canonical Wnt pathway is widely believed to drive cancer via a two-faceted mechanism (McDonald and Silver, 2009)—(1) Non-canonical Wnt signaling suppresses tumorigenesis by antagonizing the canonical β-catenin/TCF/LEF pathway, and inhibition of non-canonical Wnt signaling heralds neoplastic transformation (Ishitani et al., 2003; Medrek et al., 2009; Grumolato et al., 2010); (2) Hyperactivation of non-canonical Wnt signaling enhances cancer invasion/metastasis by activation of Rac1 and remodeling of the actin cytoskeleton (Yamamoto et al., 2009) and by upregulating CamKII and PKC (Weeraratna et al., 2002; Dissanayake et al., 2007). Little is known as to how such dysregulation of non-canonical Wnt signaling, that is, early inhibition and late hyperactivation is orchestrated during cancer progression.

Non-canonical Wnt signaling is initiated by the binding of Wnt ligands to receptors of the Frizzled (FZDR) family. These receptors belong to the G protein-coupled receptor (GPCR) superfamily, which classically activate trimeric G proteins. However, the interplay between FZDR and G proteins in Wnt signaling is very controversial—on one hand, there is a wealth of evidence indicating that trimeric G proteins regulate Wnt signaling (Malbon, 2004; Katanaev et al., 2005; Liu et al., 2005; Schulte and Bryja, 2007; Koval et al., 2011). On the other hand, definitive evidence for the direct activation of trimeric G proteins by FZDR's is elusive. The experimental difficulties and controversies in the field have led to provocative speculations that FZDRs may not bind G proteins directly, but do so indirectly via other intermediates within the Wnt signaling pathway (Schulte and Bryja, 2007), but such intermediate ‘linker’ molecules have not been identified. Recent advances in the field of trimeric G protein signaling have important implications in this regard. It has become increasingly clear that the activity of trimeric G proteins is regulated by a plethora of accessory proteins (Siderovski and Willard, 2005; Sato et al., 2006; Blumer and Lanier, 2014) beyond classical activation by GPCRs. Among these accessory proteins, a subset of proteins called non-receptor Guanine nucleotide exchange factors (GEFs) are uniquely positioned to fulfill the role of an intermediate to trigger G protein signaling upon Wnt stimulation because they are cytoplasmic factors capable of activating G proteins (Tall et al., 2003; Lanier, 2004; Natochin et al., 2005; Lee and Dohlman, 2008; Garcia-Marcos et al., 2009, 2011b; Oner et al., 2013).

Here, we identified Daple, a previously described binding partner of the Wnt signaling protein Dishevelled (Dvl) (Oshita et al., 2003; Kobayashi et al., 2005), as a non-receptor GEF for trimeric G proteins. We demonstrate that a novel G protein regulatory motif enables Daple to couple G protein activation to FZDRs, which in turn initiates non-canonical Wnt signaling pathways. We also demonstrate how bimodal dysregulation in Daple expression modulates non-canonical Wnt signaling during cancer progression.

## Results

### Daple possesses a GBA motif and binds to Gαi subunits

**Figure 1.**
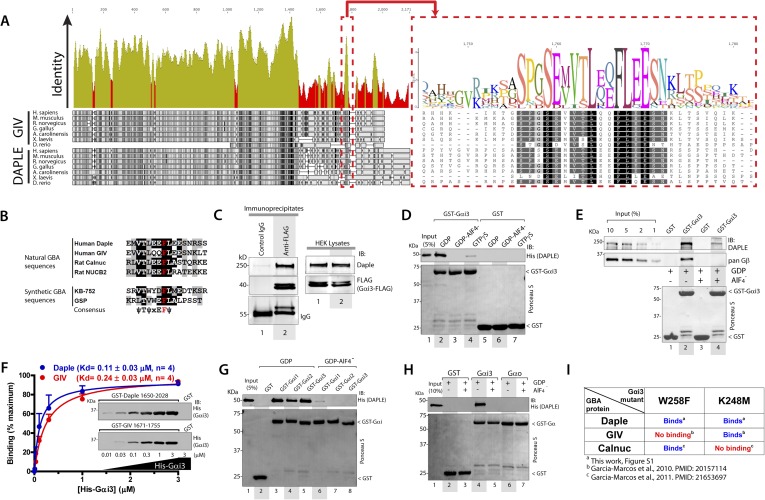
Daple contains a GBA motif. (**A**) Phylogenetic sequence analysis reveals a conserved motif in Daple similar to GIV's Gα-binding and activating (GBA) motif within an otherwise highly divergent C-terminal domain. Sequences of GIV and Daple from different species were aligned and the degree of identity at each position plotted. A high degree of identity is observed in the N-terminal region (<∼aa 1400), whereas the C-terminal domain (>aa 1400) is highly divergent. The peak of highest identity (red box) within the C-terminal domain corresponds to the GBA motif (enlarged on the right). (**B**) Daple's putative GBA motif is similar to known GBA sequences. Alignment of the putative GBA motif of Daple with the natural GBA sequences of GIV, Calnuc and NUCB2, and the synthetic GBA sequences of KB-752 and GSP peptides. Consensus is shown below (ψ = hydrophobic, x = any). (**C**) Full-length Daple binds to Gαi3 in cells. Equal aliquots of lysates of HEK293 cells expressing Gαi3-FLAG were incubated with anti-FLAG mAb or control IgG and protein G beads. Immune complexes were analyzed for Daple and Gαi3 (FLAG) by immunoblotting (IB). Gβ was monitored as positive Gαi3-binding control. (**D**) Purified Daple binds directly to inactive but not active Gαi3. Purified, recombinant GST-Gαi3 preloaded with GDP (inactive), GDP + AlF_4_^−^ (active), or GTPγS (active) and immobilized on glutathione-agarose beads was incubated with purified His-Daple-CT (aa 1650–2028, containing the putative GBA motif) as indicated. Resin-bound proteins were eluted, separated by SDS-PAGE and analyzed by Ponceau S-staining and IB with the indicated antibodies. No binding to GST alone was detected. (**E**) Full-length Daple expressed in cells binds preferentially to inactive vs active Gαi3. Purified, recombinant GST-Gαi3 preloaded with GDP (inactive) or GDP + AlF_4_^−^ (active) and immobilized on glutathione-agarose beads was incubated with cell lysates of Cos7 cells expressing full-length myc-Daple as indicated. Bound proteins were analyzed for Daple (myc) and Gβ by IB as in **D**. Binding of Gβ to inactive but not active Gαi3 was used as positive control. No binding of myc-Daple or Gβ to GST alone was detected. (**F**) Daple and GIV bind to Gαi3 with comparable submicromolar affinities. *Inset,* Purified GST-Daple-CT and GST-GIV-CT (aa 1671–1755, containing the GBA motif) immobilized on glutathione-agarose beads were incubated with increasing amounts (0.01–3 µM) of purified His-Gαi3 (GDP-loaded) and binding analyzed by IB as described in (**D**). No binding to GST alone was detected at the highest His-Gαi3 concentration tested. *Graph,* Gαi3 binding was quantified by measuring band intensities and data fitted to a single-site binding hyperbola (Daple = BLUE, GIV = RED) to determine the equilibrium dissociation constants (Kd). Mean ± S.E.M of four independent experiments. (**G**) Daple binds to all three Gαi subunits. Binding of His-Daple-CT to GST-fused Gαi1, Gαi2, or Gαi3 in the inactive or active conformations was analyzed exactly as described in (**D**). (**H**) Daple selectively binds to Gαi, but not Gαo. Binding of His-Daple-CT to GST-fused Gαi3 or Gαo in the inactive or active conformations was analyzed exactly as described in (**D**). (**I**) Daple binds to Gαi3 mutants that do not bind to other GBA proteins. Table summarizing the binding properties of Gαi3 K248M and W258F mutants to Daple (from Figure 1—figure supplement 1) and GIV or Calnuc (Garcia-Marcos et al., 2010, 2011b). **DOI:** http://dx.doi.org/10.7554/eLife.07091.003

We recently discovered the first GEF motif for trimeric G proteins, that is, the Gα-binding and activating (GBA) motif, in the C-terminal region of the non-receptor protein GIV (Garcia-Marcos et al., 2009). We showed that GIV binds and activates Gα subunits of the Gi subfamily via its GBA motif and regulates signal transduction. GIV is one of the 3 members of the CCDC88 family, which have in common an N-terminal HOOK domain followed by a long coiled-coil region but are highly divergent in their C-terminal region (Le-Niculescu et al., 2005; Enomoto et al., 2006): CCDC88b (aka GIPIE) completely lacks this C-terminal region, whereas the C-terminal region of CCDC88c (aka Daple) shows significant divergence (15% identity, 26% similarity) compared to CCDC88a's (i.e., GIV) (Figure 1A). The divergence in the C-terminal sequence allows CCDC88 proteins to associate with different proteins and regulate diverse biological processes (Le-Niculescu et al., 2005; Enomoto et al., 2006), for example, a PDZ-binding motif (PBM) is found exclusively in Daple, at its extreme C-terminus, which binds the PDZ domain of Dvl and regulates Wnt signaling (Oshita et al., 2003; Kobayashi et al., 2005). Despite these apparent sequence differences among CCDC88 family members, a more detailed analysis of the C-terminal sequences of GIV and Daple from different vertebrate species revealed a cryptic GBA motif in Daple localized within the otherwise highly divergent C-terminal region (Figure 1A). This putative GBA motif (aa 1668–1683) in Daple shares a high degree of similarity to previously reported GBA motifs found in proteins (Garcia-Marcos et al., 2009, 2011b) and synthetic peptides (Johnston et al., 2005; Austin et al., 2008) with GEF activity towards Gαi proteins (Figure 1B). As a first step to investigate the functionality of this GBA motif, we carried out co-immunoprecipitation (IP) experiments, which revealed that full-length endogenous Daple in HEK293 cells interacts with the trimeric G protein Gαi3 (Figure 1C). We next investigated if the interaction between Daple and G proteins presents the biochemical properties previously reported for other GBA motif sequences, that is, they bind directly to the G protein with submicromolar to low-micromolar affinity when it is in the inactive but not active conformation (Tall et al., 2003; Ghosh et al., 2008). Recombinant purified GST-Gαi3 bound robustly to purified His-Daple CT (aa 1650–2028, containing the GBA motif) when loaded with GDP (inactive) but not when loaded with GDP/AlF_4_^−^ or GTPγS (both mimic the GTP-bound active G protein) (Figure 1D). Equivalent results were obtained when lysates of mammalian cells expressing full-length Daple were used in the pulldown assays (Figure 1E). Binding of His-Gαi3-GDP to GST-Daple CT was saturable, and fitting of the data to a one-site binding curve revealed a submicromolar equilibrium dissociation constant (Kd = 0.11 ± 0.03 µM, n = 4), indicating a slightly higher affinity of the G protein for Daple than for GIV (Kd = 0.24 ± 0.03 µM, n = 4) (Figure 1F).

**Figure 1—figure supplement 1.**
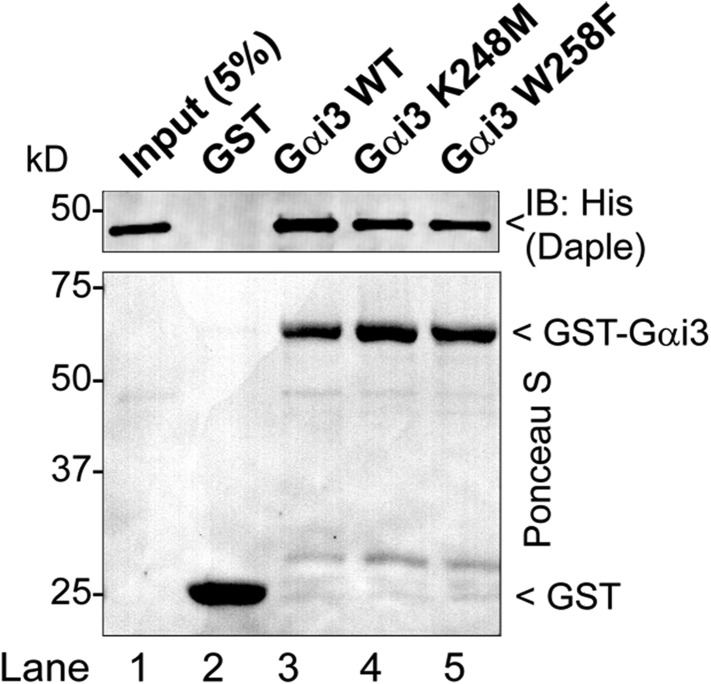
Daple binds mutants of Gαi3 that do not bind GIV (W258F) or Calnuc (K248M). Purified, recombinant GST-Gαi3 (WT and mutants) preloaded with GDP and immobilized on glutathione-agarose beads was incubated with purified His-Daple-CT (aa 1650–2028) as indicated. Resin-bound proteins were eluted, separated by SDS-PAGE, and analyzed by Ponceau S-staining and IB with anti-His antibodies. No binding to GST alone was detected. **DOI:** http://dx.doi.org/10.7554/eLife.07091.004

Another common feature among previously reported GBA motifs is their high-G protein specificity, that is, they not only bind preferentially to Gi subfamily members but can discriminate within this subfamily by binding to Gαi subunits but not to the close homologue Gαo (∼75% overall similarity to Gαi1/2/3 subunits) (Slep et al., 2008). We found that this is also the case for Daple because it interacts with Gαi1, Gαi2, and Gαi3 (although binding to Gαi2 is partially reduced compared to Gαi1 and Gαi3) (Figure 1G) but not with Gαo (Figure 1H). Despite these biochemical properties shared with related GBA motifs, we found that binding of Daple to Gαi has unique structural determinants that differentiate it from other proteins with a GBA motif, that is, GIV and Calnuc. We found that mutants of Gαi3 that were previously shown (Garcia-Marcos et al., 2010, 2011b) to be incapable of binding to GIV or Calnuc (i.e., W258F or K248M, respectively) retain their ability to bind Daple (Figure 1I, Figure 1—figure supplement 1). This result indicates that the Daple–Gαi3 interface has unique molecular features that provide specificity by making it different from other GBA motif-G protein interactions.

Taken together, these results demonstrate that Daple possesses a GBA motif, and that its interaction with G proteins presents all the biochemical features, that is, G protein activation status dependence, affinity and specificity, characteristic of a GBA motif-containing protein.

### Identification of critical structural determinants for the interaction between Gαi and Daple's GBA motif

**Figure 2.**
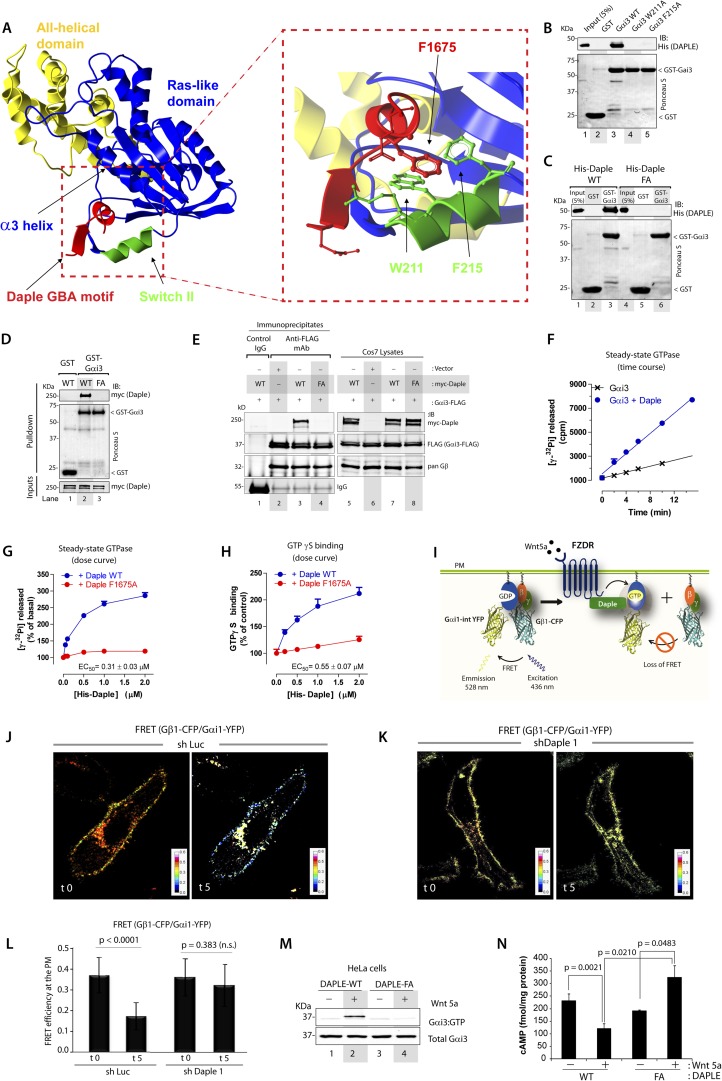
Daple binds and activates Gαi3 in vitro and in vivo via its GBA motif. (**A**) Prediction of molecular contacts critical for the Daple-Gαi interaction. Homology-based model of Daple's GBA motif (Red) bound to Gαi3 (green = Switch II, blue = ras-like domain, yellow, all-helical domain) with an enlarged section depicting a putative hydrophobic contact between Daple's F1675 and Gαi3's W211/F215. (**B**) Mutation of residues in the SWII region of Gαi3 disrupts Daple binding. Binding of His-Daple-CT to GST-Gαi3 WT, W211A, or F215A was analyzed exactly as described in Figure 1D. (**C**) Mutation of Daple F1675 to A abrogates Gαi3 binding. Binding of His-Daple-CT WT or F1675A (FA) to GST-Gαi3 was analyzed exactly as described in Figure 1D. (**D**) F1675A mutation disrupts binding of full-length Daple expressed in cells to Gαi3. Myc-Daple WT or F1675A (FA) was expressed in Cos7 cells and binding to GST-Gαi3 analyzed exactly as described in Figure 1E. (**E**) Binding of full-length Daple to Gαi3 in cells is abolished upon F1675A mutation. Lysates of Cos7 cells expressing Gαi3-FLAG and myc-Daple-WT or F1675A (FA) were incubated with anti-FLAG mAb and subsequently with protein G beads. Immune complexes were analyzed for Daple (myc) and Gαi3 (FLAG) by IB. Gβ was monitored as positive Gαi3-binding control. (**F**) Daple accelerates the rate of Gαi3 steady-state GTPase activity. The steady-state GTPase activity of His-Gαi3 alone (black) or in the presence of 2 µM His-Daple-CT (blue) was determined by measuring the production of [^32^P]Pi at different time points as described in ‘Materials and methods’. One experiment representative of 3 is shown. (**G**) Daple WT but not F1675A (FA) accelerates the rate of Gαi3 steady-state GTPase activity in a dose-dependent manner. The steady-state GTPase activity of His-Gαi3 was determined in the presence of increasing concentrations (0–2 µM) of His-Daple-CT WT (blue) or His-Daple-CT FA (red) by measuring the production of [^32^P]Pi at 15 min. Mean ± S.E.M of five independent experiments. (**H**) Daple WT but not F1675A dose-dependently accelerates the rate of GTPγS binding to Gαi3. GTPγ^35^S binding to His-Gαi3 at 15 min was determined in the presence of increasing concentrations (0–2 µM) of His-Daple-CT WT (blue) or His-Daple-CT FA (red). Mean ± S.E.M of four independent experiments. (**I**) Schematic for the Gαi1-intYFP and Gβ1-CFP constructs used as paired Fӧrster resonance energy transfer (FRET) probes in **J**, **K**, and **L**. (**J**–**L**) Heterotrimers of Gi1 (Gαi1 and Gβ1γ2) are dissociated at the plasma membrane (PM) in control (**J**, sh Luc), but not Daple-depleted (**K**, sh Daple 1) HeLa cells after Wnt5a stimulation. Control (Left) or Daple-depleted (Right) HeLa cells (sh Daple 1 described in Figure 2—figure supplement 1A,B) cotransfected with Gαi1-intYFP, Gβ1-CFP, and Gγ2 were maintained overnight in 0.2% FBS and subsequently stimulated with 0.1 mg/ml Wnt5a and analyzed for FRET by confocal microscopy. Representative freeze-frame images from live-cell movies are shown, which display intensities of acceptor emission due to FRET in each pixel. Activation of Gi, as determined by the loss of interaction (i.e., FRET) between Gαi1 and Gβ1γ2 was observed exclusively after ligand stimulation (compare t0 and t5) in control (**J**), but not in Daple-depleted HeLa cells (**K**). (**L**) Bar graphs display differences between FRET intensities observed in control vs Daple-depleted cells in (**J**, **K**). Error bars representing mean ± S.D. of 5 randomly chosen regions of interest (ROIs) at the PM per cell, from 4 to 5 cells per experiment, from three independent experiments. (**M**) HeLa cells expressing Daple-WT, but not Daple-F1675A activate Gαi3 in response to Wnt5a stimulation, as determined by immunoprecipitation (IP) with conformationally-sensitive anti-Gαi:GTP antibodies. Daple-depleted HeLa cells transiently transfected with myc-Daple WT or F1675A (FA) were serum-starved and treated (+) or not (−) with 0.1 mg/ml Wnt5a for 20 min were subjected to immunoprecipitation with antibodies that selectively recognize active Gαi subunits in their GTP-bound state. Immune complexes (top) and lysates (bottom) were analyzed for active Gαi3:GTP and total Gαi3 by immunoblotting (IB) with anti-Gαi3 antibody. (**N**) HeLa cells expressing Daple-WT, but not Daple-F1675A inhibit cAMP in response to Wnt5a stimulation, as determined by radioimmunoassay. HeLa cells transiently transfected with myc-Daple WT or F1675A (FA) incubated with forskolin and PDE inhibitors for 10 min, treated (+) or not (−) with 0.1 mg/ml Wnt5a for 20 min and cAMP levels quantified as detailed in ‘Materials and methods’. Mean ± S.D. of three independent experiments. **DOI:** http://dx.doi.org/10.7554/eLife.07091.005

To gain insights into the interface between Daple and Gαi proteins, we took advantage of the previously published atomic structure of KB-752, a synthetic GEF peptide similar to the GBA motif (Figure 1A), in complex with Gαi1 (Johnston et al., 2005). We used this structure as a template to build a homology model of the complex between the GBA motif of Daple and Gαi3 (Figure 2A). Our first prediction based on this model was that Daple would bind to a hydrophobic cleft on the G protein located between the switch II (SwII) region and the α3 helix. This seemed to be the case because two molecules known to bind onto the SwII/α3 cleft, that is, the synthetic GEF peptide KB-752 (Figure 2—figure supplement 1A) and His-GIV-CT (aa 1660–1870, containing its GEF motif) (Figure 2—figure supplement 1B), competed with His-Daple-CT for binding to GST-Gαi3. We further substantiated the identity of the binding pocket using site-directed mutagenesis. Analysis of our homology model suggested that a major molecular contact is established by the hydrophobic interaction between the aromatic residues W211 and F215 located in the SwII region of Gαi3 and Daple's F1675 (Figure 2A). Binding of His-Daple-CT to GST-Gαi3 was dramatically impaired upon mutation of W211 or F215 to Alanine (Ala; A) (Figure 2B), indicating that these hydrophobic residues of the SwII/α3 cleft serve as a docking site for Daple. Importantly, W211A and F215A mutations have been previously shown not to disturb the native biochemical properties of Gαi proteins (Thomas et al., 2004), and therefore, their inability to bind Daple is not a consequence of an overall defect in G protein folding or function. Furthermore, mutation of Daple's F1675, the residue in its GBA motif predicted to interact with W211 and F215 of the G protein (Figure 2A) to Ala abolished GST-Gαi3 binding to either recombinant His-Daple-CT (Figure 2C) or full-length myc-Daple expressed in mammalian cells (Figure 2D). Equivalent results were obtained in co-IP experiments in that binding of full-length myc-Daple and Gαi3 co-expressed in mammalian cells was dramatically impaired upon mutation of F1675 to A (Figure 2E; henceforth referred to as FA). Taken together, these results demonstrate that Daple utilizes its GBA motif to bind onto the SwII/α3 hydrophobic cleft of Gαi3.

**Figure 2—figure supplement 1.**
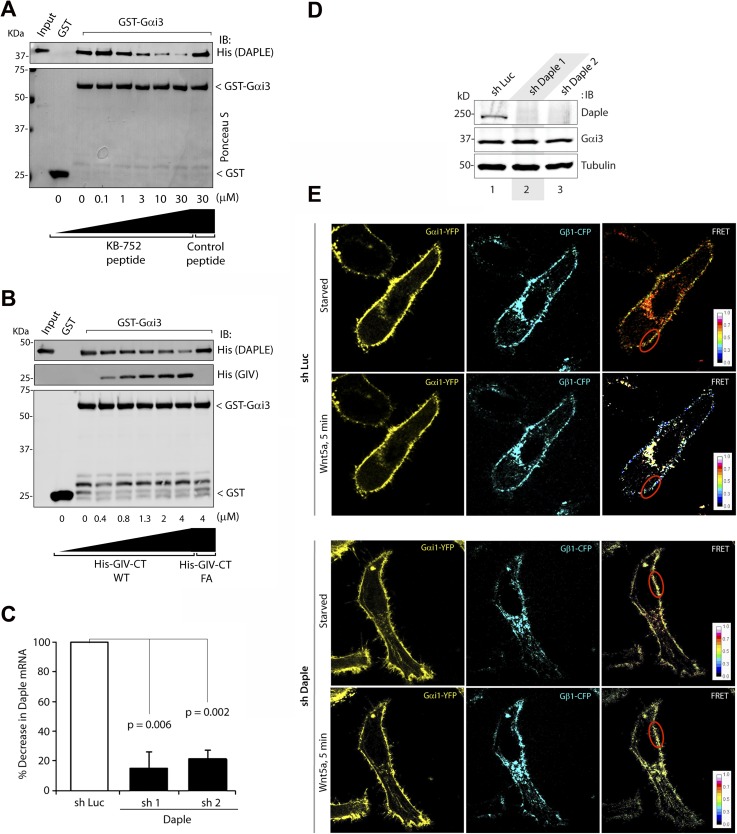
Binding of Daple to Gαi triggers activation of Gi at the PM after Wnt5a stimulation. (**A**, **B**) Daple competes for binding to Gαi3 with peptides/proteins that dock onto the switchII/α3 cleft of the G protein. (**A**) Purified, recombinant GST-Gαi3 preloaded with GDP and immobilized on glutathione-agarose beads (∼0.3 mM) was incubated with a fixed concentration (∼0.2 mM) of purified His-Daple-CT (aa 1650–2028) in the presence of the indicated concentrations of KB-752 or a control peptide. Resin-bound proteins were eluted, separated by SDS-PAGE, and analyzed by Ponceau S-staining and IB with anti-His antibodies. No binding to GST alone was detected. One experiment representative of 3 is shown. (**B**) Analogous experiments to those described in **A** were carried out using His-GIV-CT WT and His-GIV-CT F1685A (as negative control) instead of peptides. (**C**–**E**) Daple is essential for activation of trimeric Gi at the PM after Wnt5a stimulation. (**C**, **D**) Two independent shRNA sequences targeting the 3′ UTR of the gene efficiently (<90%) deplete Daple mRNA (**A**) and protein (**B**) from HeLa cells. The knock-down efficiency was assessed by comparing Daple mRNA by quantitative PCR (qPCR) (**C**) or protein by IB (**D**) on HeLa cells stably expressing two Daple-targeting (shDaple1 and shDaple2) or control (shRNA targeting luciferase [shLuc]) shRNA sequences. (**E**) Control (Luc shRNA) or Daple-depleted (Daple shRNA1) HeLa cells co-transfected with Gαi1-YFP, Gβ1-CFP, and Gγ2 were starved overnight in media containing 0.2% FBS prior to stimulation with Wnt5a and analyzed for FRET using confocal microscope. Representative freeze-frame images from live-cell movies are shown, which display acceptor (Gαi1-YFP), donor (Gβ1-CFP) and intensities of acceptor emission due to FRET in each pixel (from left to right). Activation of Gi, as determined by the loss of interaction (i.e., FRET efficiency) between Gαi1-YFP and Gβ1-CFP is observed at the PM exclusively after ligand stimulation (compare t0 and t5) in Luc shRNA treated control cells, but not in Daple-depleted cells. Red circle = ROI at the PM. **DOI:** http://dx.doi.org/10.7554/eLife.07091.006

### Daple is a *bona fide* GEF for Gαi in vitro

GEFs are defined by their ability to accelerate the rate of nucleotide exchange. To determine if binding of Daple to Gαi3 accelerates the rate of nucleotide exchange on the G protein, we carried out two well-established enzymatic assays—the steady-state GTPase assay, which indirectly reflects the rate of nucleotide exchange (Mukhopadhyay and Ross, 2002), and the GTPγS-binding assay, which directly measures the rate of nucleotide exchange. We found that incubation of His-Gαi3 with His-Daple-CT accelerated the rate of steady-state GTP hydrolysis ∼threefold over the basal activity (Figure 2F). This acceleration of Gαi3 steady-state GTPase activity by Daple was dose-dependent, with an EC_50_ of 0.25 ± 0.06 µM (similar to the estimated Kd for the Daple–Gαi3 interaction, Figure 1F), and was greatly diminished (>90%) in parallel reactions in which His-Daple-CT WT was replaced by the Gαi3 binding-deficient mutant F1675A (Figure 2G). We further validated that Daple is a *bona fide* GEF for Gαi using GTPγS-binding assays, which showed that the initial rate of nucleotide binding by His-Gαi3 was increased by His-Daple-CT in a dose-dependent manner, but it was not significantly affected by His-Daple-CT FA (Figure 2H). Thus, Daple activates Gαi proteins in vitro by virtue of a GEF activity associated to its GBA motif.

### Daple activates Gαi in cells responding to Wnt5a

Next, we asked whether Daple activates G proteins in mammalian cells responding to Wnt5a. To this end, we generated HeLa cells stably expressing Daple-targeting shRNA sequences under the control Cre recombinase activity (see Supplemental Materials for the rationale behind the choice of this cell type and others in subsequent sections). Upon Cre treatment, two independent shRNA sequences reduced Daple mRNA levels by >80% (Figure 2—figure supplement 1C) and the Daple protein to virtually undetectable levels (Figure 2—figure supplement 1D) compared to cells expressing a control shRNA targeting luciferase (shLuc). We used these cells in a previously validated assay in which activation of Gi is monitored by dissociation of fluorescently tagged Gαi and Gβγ subunits with a resultant loss of Förster resonance energy transfer (FRET) (Janetopoulos et al., 2001; Bunemann et al., 2003; Gibson and Gilman, 2006) (Figure 2I–L). When control HeLa cells co-expressing Gαi1-YFP (internal tag), CFP-Gβ_1_ (N-terminal tag), and Gγ_2_ (untagged) were stimulated with Wnt5a, we observed a significant loss of FRET, that is, Gi heterotrimer dissociated into Gαi-YFP and CFP-Gβγ subunits at the plasma membrane (PM) within 5 min as determined by a significant drop in FRET efficiency from 0.36 ± 0.08 to 0.17 ± 0.06 (Figure 2J,L, Figure 2—figure supplement 1E), indicating that Gi is activated in response to Wnt5a. No significant drop in FRET was observed in Daple-depleted cells (Figure 2K,L; Figure 2—figure supplement 1E), indicating that donor-CFP-Gβγ and acceptor-Gαi-YFP subunits continued to interact (i.e., Gi heterotrimers remained intact) at the PM regardless of Wnt5a stimulation, and that Gαi remained inactive. These results demonstrate that Daple is essential for activation of Gi upon Wnt5a stimulation.

Next, we asked if the GBA motif in Daple is essential for activation of Gαi in cells responding to Wnt5a. To this end, we analyzed activation of Gαi in HeLa cells expressing Daple-WT or FA using an anti-Gαi:GTP mAb that specifically recognizes Gαi in a GTP-bound active conformation (Lane et al., 2008a). Previous work by others (Lane et al., 2008a) and by us (Lopez-Sanchez et al., 2014) has demonstrated that this antibody can specifically recognize active Gαi in cells. When we immunoprecipitated Gαi from HeLa cells, active Gαi3 was immunoprecipitated exclusively after Wnt5a stimulation in cells expressing Daple-WT (Figure 2M), but not in those expressing Daple-FA. These results indicate that an intact GBA motif is essential for Daple to activate Gαi3 after Wnt5a stimulation. To further substantiate this, we determined the intracellular levels of cAMP as a measure of the activity of adenylyl cyclase, which is directly inhibited by active Gαi subunits. We found that Wnt5a stimulation suppressed cAMP levels by ∼50% in HeLa cells expressing Daple-WT, but no such suppression occurred in cells expressing Daple-FA (Figure 2N). Taken together, these results demonstrate that Daple is a *bona fide* GEF that activates Gαi proteins in vitro and in cells responding to Wnt5a via its GBA motif.

### Daple activates Rac1 and PI3K-Akt signaling via release of free Gβγ subunits

**Figure 3.**
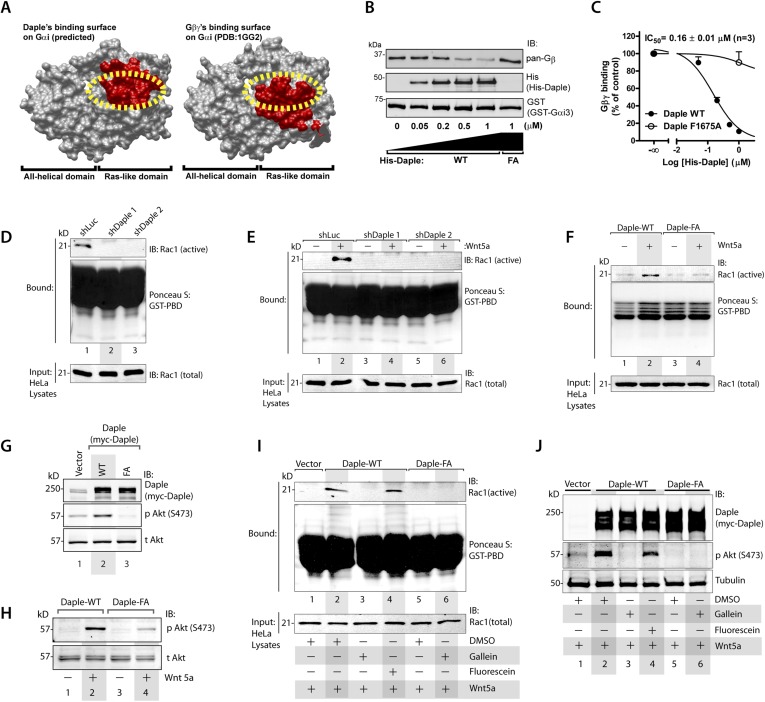
Daple's GBA motif triggers the release of ‘free’ Gβγ subunits, which in turn enhance Rac1 and PI3K-Akt signaling. (**A**) Daple's GBA motif and Gβγ subunits are predicted to dock onto an overlapping binding site on Gαi. Binding areas (in red) for Daple (left) or Gβγ (right) on Gαi (solid gray) were extracted from a homology-based model of Daple-Gαi3 and the crystal structure of the Gαi1·Gβγ complex (Protein Data Bank [PDB]: 1GG2), respectively. (**B**, **C**) Daple displaces Gβγ subunits from Gαi3 via its GBA motif. GST-Gαi3·Gβγ preformed complexes immobilized on glutathione beads were incubated with increasing concentrations of His-Daple-CT WT or F1675A (FA). Bound proteins were analyzed by IB (**B**) and Gβγ binding data fitted to a single-site competition curve (**C**). Mean ± S.E.M. of three independent experiments. (**D**, **E**) Activation of Rac1 is impaired in Daple-depleted HeLa cells. Control (shLuc) or two clones of Daple-depleted HeLa cell lines (sh Daple 1 and 2) (described in Figure 2—figure supplement 1A,B) were incubated in 2% serum media (**D**) or starved and treated (+) or not (−) with Wnt5a (0.1 mg/ml) for 5 min (**E**) and analyzed for Rac1 activation by pulldown assays using GST-PBD. (**F**) Activation of Rac1 is impaired in cells expressing Daple-F1675A (FA) mutant compared to those expressing Daple-WT. Daple-depleted (sh Daple 1) HeLa cells transiently transfected with myc-Daple-WT or FA were starved and stimulated with Wnt5a and analyzed for Rac1 activation as in **E**. (**G**, **H**) Daple's GBA motif is required for activation of PI3K-Akt signaling in HeLa cells, as determined by phosphorylation of Akt at S473. Daple-depleted (sh Daple 1) HeLa cells transiently transfected with myc-Daple WT or F1675A (FA) were incubated in a 2% serum media (**G**) or in a 0.2% serum media overnight and treated (+) or not (−) with 0.1 mg/ml Wnt5a for 5 min (**H**) prior to lysis. Equal aliquots of whole-cell lysates were analyzed for Akt phosphorylation (pAkt S473) by IB. (**I**, **J**) Inhibition of Gβγ signaling impairs Daple-dependent activation of Rac1 and Akt. Daple-depleted (sh Daple 1) HeLa cells transiently transfected with myc-Daple WT were treated with DMSO, 10 µM of the Gβγ inhibitor gallein or its inactive analog fluorescein for 6 hr, as indicated, and analyzed for Rac1 (**I**) or Akt (**J**) activation by IB or pulldown assays, respectively. **DOI:** http://dx.doi.org/10.7554/eLife.07091.007

In addition to modulation of cellular cAMP, another major consequence of activating Gαi subunits is the release of free Gβγ subunits, which in turn modulates a wide array of signaling pathways (Smrcka, 2008, 2013). Comparative analysis of the crystal structure of the Gαi1·βγ trimer and the homology model of Daple's GBA motif bound to Gαi3 revealed that Gβγ and Daple have overlapping binding sites on Gαi subunits (Figure 3A). Based on this, we reasoned that binding of Daple to Gαi will displace Gβγ from trimeric Gαi·βγ complexes. We found that is indeed the case because His-Daple-CT WT, but not the FA mutant (which cannot bind Gαi), displaced Gβγ from a pre-assembled complex with GST-Gαi3 (Figure 3B). The IC_50_ for this displacement was 0.16 ± 0.01 µM (Figure 3C), which is consistent with the estimated affinity of Daple for Gαi3 (Figure 1F).

To determine if the ‘free’ Gβγ released by Daple's GBA motif modulated cellular signaling, we analyzed two signaling pathways, Rac1 and PI3K-Akt because previous studies have demonstrated a direct and critical role of ‘free’ Gβγ subunits in enhancement of these signals (Leopoldt et al., 1998; Welch et al., 2002; Niu et al., 2003; Ueda et al., 2008; Xu et al., 2012), and because they represent major signals downstream of the non-canonical Wnt pathway (Kawasaki et al., 2007; Nishita et al., 2010; Anastas et al., 2014). Rac1 activity, as determined in pulldown assays using the p21 binding domain (PBD) of PAK1 (Knaus et al., 2007), was suppressed in Daple-depleted HeLa cells both at steady-state in the presence of low serum (Figure 3D) as well as after Wnt5a stimulation (Figure 3E). Furthermore, Wnt5a triggered activation of Rac1 in cells expressing Daple-WT, but not the FA mutant (Figure 3F). These findings indicate that Daple and its GBA motif are required for the efficient activation of Rac1 activity. Similarly, we found that activation of Akt, as determined by phosphorylation of the kinase at Ser473 was enhanced in cells expressing Daple WT, but not the FA mutant, both at steady-state in the presence of low serum (Figure 3G), as well as after Wnt5a stimulation (Figure 3H), indicating that Daple's GBA motif is essential for enhancement of PI3K/Akt signaling.

To pinpoint whether the enhanced Rac1 and Akt signals are triggered directly by ‘free’ Gβγ subunits that are released by Daple, we used a Gβγ inhibitor, that is, Gallein, that selectively blocks the interaction between Gβγ with key downstream effectors (Bonacci et al., 2006; Lehmann et al., 2008; Smrcka et al., 2008; Urano et al., 2008; Seneviratne et al., 2011). We found that incubation of HeLa cells expressing Daple WT with Gallein effectively inhibited both Rac1 (Figure 3I) and Akt (Figure 3J) activities to levels observed in cells expressing Daple FA, whereas the inactive analog, Fluorescein had no such effect. These results indicate that Daple enhances Rac1 and Akt signaling at least in part by facilitating the release of ‘free’ Gβγ subunits, which subsequently trigger signaling via downstream intermediates.

In summary, these results indicate that the dissociation of Gαi·βγ heterotrimers triggered upon Wnt5a stimulation by Daple's GBA motif sets off at least two major immediate events within the G protein signaling cascade—(1) GTP-loading of Gαi subunits, which subsequently inhibits the adenylyl cyclase/cAMP pathway and (2) release of Gβγ subunits that trigger the activation of non-canonical Wnt signaling pathways including Rac1 and PI3K-Akt.

### Daple links G proteins to ligand-activated FZDRs via its GBA motif

**Figure 4.**
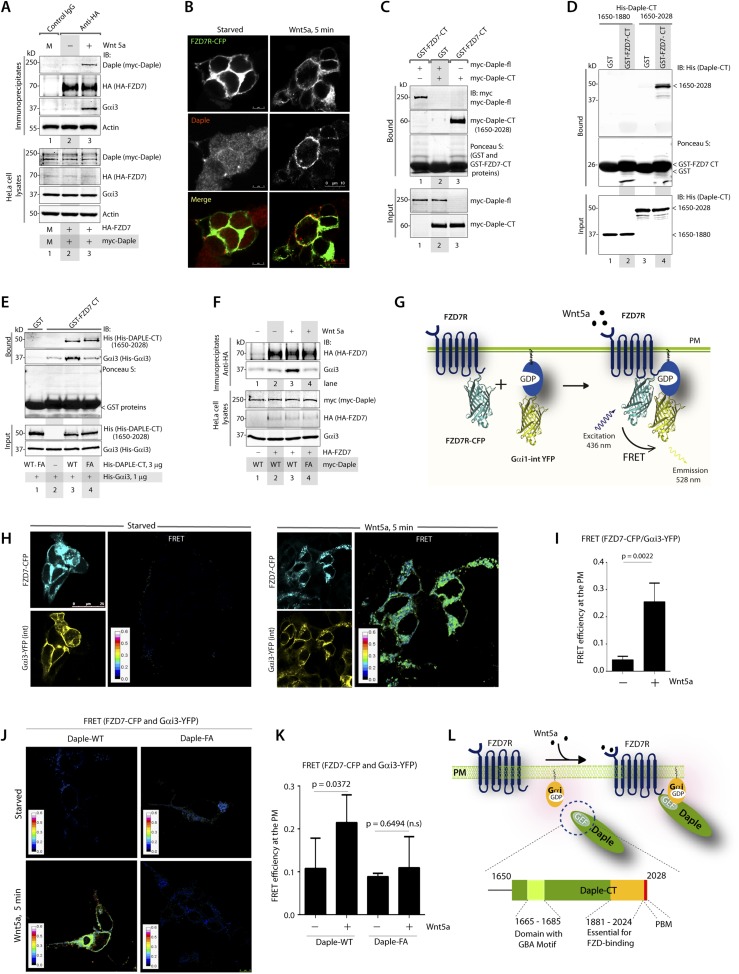
The C-terminus of Daple directly binds ligand-activated FZDRs and triggers the assembly of FZDR-Gαi complexes at the PM. (**A**) Daple and Gαi3 co-immunoprecipitate with FZD7R after Wnt5a stimulation. HeLa cells cotransfected with myc-Daple WT and HA-FZD7 were starved and stimulated with Wnt5a as in 3G. Equal aliquots of lysates (bottom) were then incubated with anti-HA mAb and subsequently with protein G beads. Immune complexes (top) were analyzed for myc (myc-Daple) and endogenous Gαi3 by IB. (**B**) Daple is recruited to the PM after Wnt5a stimulation, where it colocalizes with FZD7R. HEK293 cells expressing FZD7-CFP (pseudocolored green) were grown on cover slips coated with Poly-D-Lysine, starved for 24 hr (0% FBS) and treated with 0.1 mg/ml Wnt5a as in 4A. Cells were fixed and stained for Daple (red) and analyzed by confocal microscopy. (**C**) The C-terminal region (1650–2028 aa) is sufficient for Daple to bind FZD7R. Lysates of Cos7 cells expressing full-lenght myc-Daple-WT or myc-Daple-CT (1650–2028 aa) were incubated with recombinant GST-FZD7-CT immobilized on glutathione-agarose beads in pulldown assays. Bound Daple (myc) was analyzed by IB. (**D**) Daple directly binds FZD7R and the extreme C-terminus (1881–2028) is essential for the interaction. His-Daple-CT (1650–2028 aa) or a shorter fragment of Daple-CT (1650–1880 aa) was incubated in pulldown assays with immobilized GST-FZD7-CT exactly as above. Bound Daple-CT (His) was analyzed by IB. (**E**) Daple's GBA motif is required for enhanced binding of Gαi3 to cytoplasmic tails of FZD7R in vitro. His-Gαi3 preloaded with GDP was incubated with immobilized GST-FZD7-CT, either alone (lane 2) or in the presence of His-Daple-CT (1650–2028 aa) WT (lane 3) or FA (lane 4) in pulldown assays as described in **D**. Bound Gαi3 and Daple-CT were detected by IB. (**F**) Daple's GBA motif is essential for the co-IP of Gαi3 with ligand-activated FZD7Rs. HeLa cells cotransfected with HA-FZD7 and myc-Daple-WT or FA were starved and subsequently stimulated with Wnt5a prior to lysis as in **A**. Equal aliquots of lysates (bottom) were incubated with anti-HA antibodies and subsequently with protein G beads. Immune complexes were analyzed for the presence of Gαi3 by IB. (**G**–**I**) Wnt5a stimulates formation of FZD7R-Gαi3 complexes at the PM in HEK293T cells. (**G**) Schematic of the FRET probes used in **H**. (**H**) HEK293 cells were cotransfected with FZD7-CFP and Gαi3-YFP, starved, and subsequently stimulated with Wnt5a and analyzed for FRET using confocal microscopy. Image panels display CFP, YFP, and intensities of acceptor emission due to FRET in each pixel. FRET was observed after Wnt5a stimulation (right). (**I**) Bar graphs display FRET efficiency observed at the PM in starved vs Wnt5a stimulated cells in **H**. Error bars represent mean ± S.D. The analysis represents 5 randomly chosen ROIs at the PM per cell, from 4 to 5 cells per experiment, from three independent experiments. (**J**, **K**) Daple's GBA motif is essential for the assembly of FZD7R-Gαi3 complexes at the PM. HEK293T cells were cotransfected with FZD7-CFP, Gαi3-YFP and myc-Daple (WT or FA), starved, and subsequently stimulated with Wnt5a prior to fixation. Fixed cells were stained for Daple (632 nm, far red; see Figure 4—figure supplement 2) and analyzed for FRET using confocal microscope. Image panels display the intensities of acceptor emission due to FRET in each pixel. FRET was observed in cells expressing Daple-WT, but not in cells expressing Daple-FA. (**K**) Bar graphs display the FRET efficacy observed in Daple WT vs Daple FA cells before (−) and after (+) Wnt5a stimulation. Error bars representing mean ± S.D. The analysis was done exactly as in **H**, **I**. (**L**) Schematic summary. Upon stimulation with Wnt5a, Daple's C-terminus enables the formation of FZD7R-Daple-Gαi3 complexes at the PM. Two distinct interaction modules present in-tandem within the C-terminus of Daple, the GBA motif, and the FZD-binding domain are essential for the formation of such complexes. **DOI:** http://dx.doi.org/10.7554/eLife.07091.008

Because Daple enhances non-canonical Wnt signaling that is initiated by FZDRs, we asked how Daple may modulate signals downstream of these receptors and wondered if they interact. We tested several purified GST-tagged FZDR cytoplasmic tail proteins for their ability to bind Daple from Cos7 lysates (Figure 4—figure supplement 1A–C). More specifically, we tested FZDRs 1–7, which belong to 3 evolutionary distinct subfamilies within the FZDR superfamily (Figure 4—figure supplement 1A) containing divergent sequences in their C-terminus that determine which regulatory proteins are assembled (Schulte, 2010; Dijksterhuis et al., 2014). Daple bound robustly to FZD7R, and only weakly to others, indicating that Daple may engage preferentially with FZD7R (Figure 4—figure supplement 1B,C). Based on this result, we used FZD7R in all subsequent assays to further analyze the interaction between Daple and FZDR. We found that both endogenous and exogenously expressed Daple and Gαi3 co-immunoprecipitated with FZD7R exclusively after Wnt5a stimulation (Figure 4A, Figure 4—figure supplement 2A), indicating that Daple and Gαi3 form complexes with ligand-activated FZD7R. Immunofluorescence studies revealed that in starved HEK293 cells, Daple is cytosolic in distribution, but in cells responding to Wnt5a Daple is localized at the PM, where it colocalized with FZD7R (Figure 4B). These findings suggest that the ligand-dependent interaction between FZD7R and Daple we see in 4A occurs at the PM.

**Figure 4—figure supplement 1.**
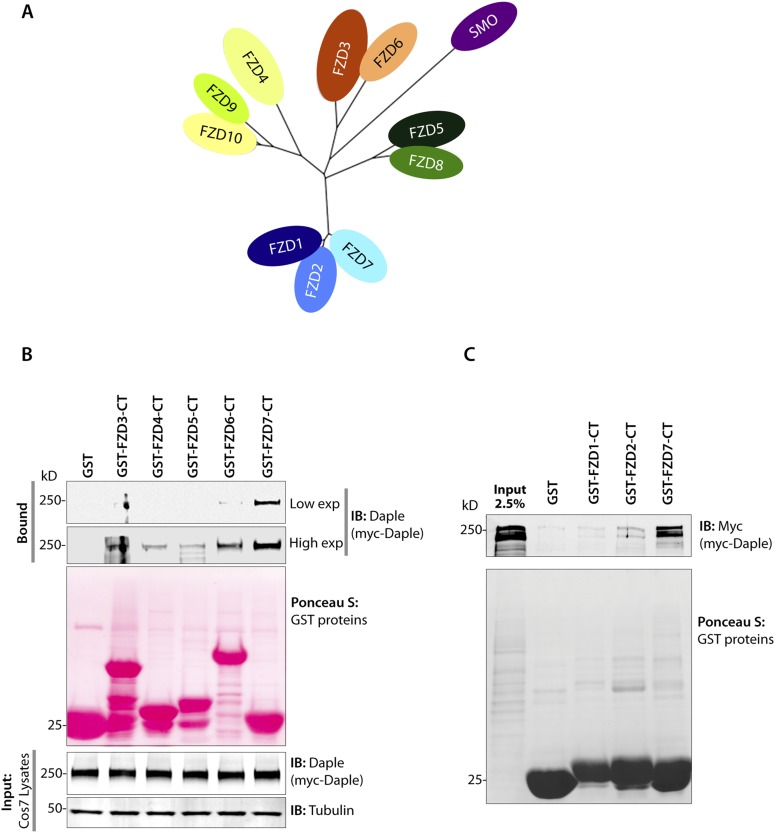
Daple preferentially binds the cytoplasmic tail of the FZD7R. (**A**) A sequence homology-based cluster tree of vertebrate Frizzled receptors (FZDRs) is shown. The FZD (IUPHAR nomenclature) family roughly clusters into four distinct families based on sequence identity (modified from Verkaar and Zaman, 2010): (I) FZD 1, 2, and 7; (II) Frizzled-5 and Frizzled-8; (III) Frizzled-3 and Frizzled-6; (IV) Frizzled-4, Frizzled-9, and Frizzled-10. The Smoothened (SMO) receptor is a distant relative of FZD receptors that functions in Hedgehog signal transduction. (**B**, **C**) Lysates of cells expressing myc-Daple full length was used as source of Daple in pulldown assays with immobilized recombinant GST-tagged C-termini of various FZDRs. Bound proteins were analyzed for Daple by IB. Full-length Daple binds preferentially to the cytoplasmic tail of FZD7R, to an intermediate extent to the cytoplasmic tail FZD6R and only weakly the cytoplasmic tails of other FZDRs. **DOI:** http://dx.doi.org/10.7554/eLife.07091.009

**Figure 4—figure supplement 2.**
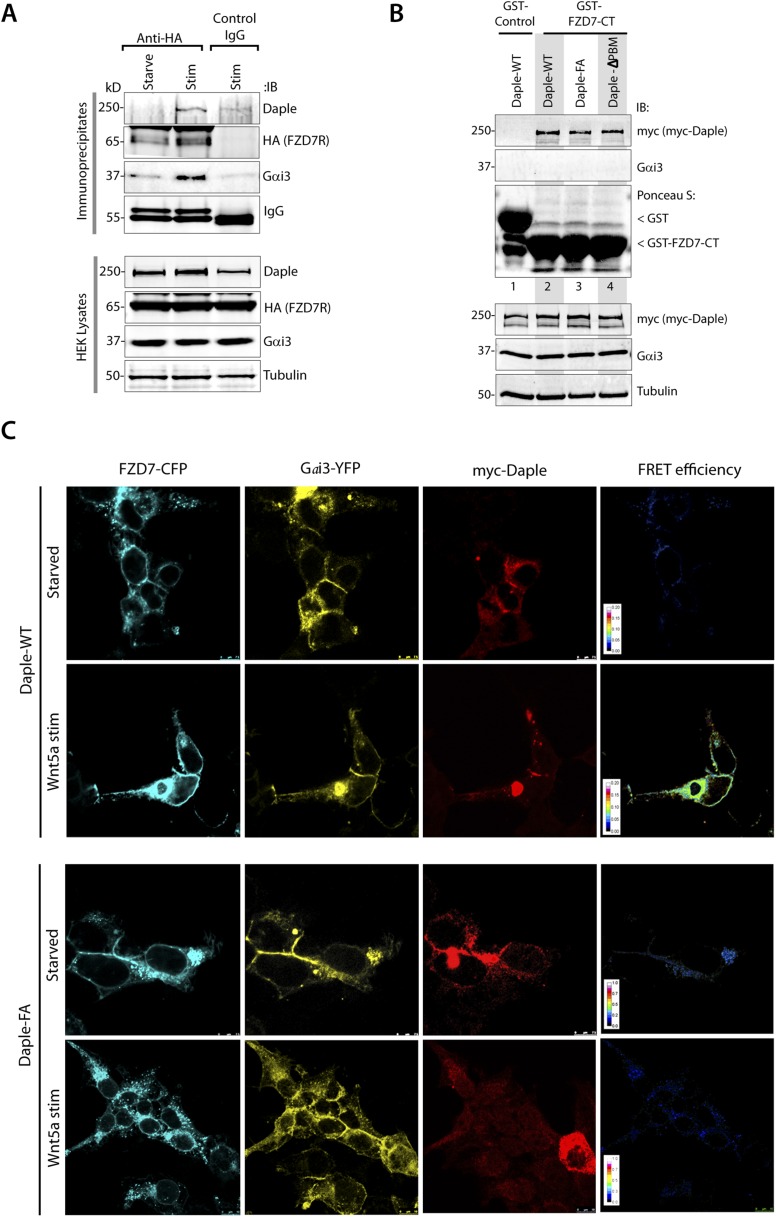
Daple binds to the C-terminus of FZD7R and links Gαi to ligand-activated receptors. (**A**) HEK cells expressing HA-tagged FZD7R were starved for 24 hr (0% FBS) and stimulated with Wnt5a for 5 min as indicated prior to lysis. IP was carried out on lysates with anti-HA or control mouse IgGs and protein G beads. Equal aliquots of lysates (bottom) and immune complexes (top) were analyzed for Daple, Gαi3, FZD7R (HA), and tubulin by IB. Endogenous Daple and Gαi3 are recruited to FZD7R exclusively after Wnt5a stimulation. (**B**) Lysates of Cos7 cells expressing myc-tagged Daple-WT or GBA-deficient (FA) and PBM-deficient (ΔPBM) mutants were used as source of Daple in pulldown assays with GST-FZD7-CT immobilized on glutathione beads. Bound proteins were analyzed for Daple by IB. Mutant Daple proteins bound FZD7 as efficiently as Daple-WT. (**C**) HEK cells co-transfected with FZD7-CFP, Gαi3-YFP, and Daple-WT or FA were starved and subsequently stimulated with Wnt5a and analyzed for FRET using confocal microscope. Representative freeze-frame images from live-cell movies are shown, which display (from left to right) donor (FZD7-CFP), acceptor (Gαi3-YFP), Daple (far-red; 632 nm) and intensities of acceptor emission due to FRET in each pixel. Interaction (i.e., FRET) is observed exclusively after Wnt5a stimulation in cells expressing Daple-WT, but not Daple-FA. **DOI:** http://dx.doi.org/10.7554/eLife.07091.010

Next, we asked which region of Daple interacts with FZD7R and whether the binding is direct. We found that the C-terminal ∼380 aa of Daple (aa 1650–2028) was sufficient to interact with GST-FZD7R-CT as efficiently as the full-length Daple (Figure 4C). Pulldown assays with the purified, recombinant His-tagged identical segment (aa 1650–2028) of Daple revealed that the binding is direct (Figure 4D). A shorter C-terminal fragment of Daple (aa 1650–1880), which lacks the ∼150 aa at the extreme C-terminus does not (Figure 4D). Furthermore, the GEF-deficient (FA) and the ΔPBM-deficient mutants bound GST-FZD7R-CT as efficiently as Daple WT (Figure 4—figure supplement 2B). These findings demonstrate that—(1) the FZD7R-Daple interaction is direct; (2) that the aa 1650–2028 in the C-terminus of Daple is sufficient to mediate the interaction; (3) that the extreme C-terminal ∼150 aa within the C-terminus (1881–2029) is essential for the interaction, whereas both the GBA and PBM motifs are dispensable.

Because Gαi3 co-immunoprecipitated with ligand-activated FZD7R-Daple complexes (Figure 4A), we asked if the interaction observed is direct or mediated by Daple. We first carried out GST pulldown assays with recombinant His-Gαi3 and the GST-tagged cytoplasmic tail of FZD7R. We found that Gαi3 bound weakly to GST-FZD7R-CT (Figure 4E; lane 2); however, binding was increased ∼fivefold in the presence of recombinant Daple-CT WT, but not the FA mutant. This raised the possibility that the ligand-dependent interaction between Gαi and FZD7 we see in cells (Figure 4A) is indirect and mediated by the GBA motif in Daple. Indeed, ligand-dependent recruitment of Gαi3 to FZD7R occurred exclusively in cells expressing full-length Daple-WT (where GBA motif is intact), but not the FA mutant (Figure 4F). Next, the spatiotemporal dynamics of ligand-dependent complex formation between FZD7R and Gαi3 was analyzed in HEK293 cells by FRET imaging (Figure 4G). We found that the probe-pair FZD7R-CFP and Gαi3-YFP interact at the PM within 5 min after ligand stimulation (FRET efficiency = 0.25 ± 0.06) (Figure 4H,I). No such interaction was observed in starved cells (FRET efficiency = 0.04 ± 0.01), demonstrating that Wnt5a triggers the assembly of complexes between ligand-activated FZD7R and Gαi3 at the PM. Furthermore, ligand-dependent assembly of such complexes occurred in cells expressing Daple-WT, but not the FA mutant (Figure 4J,K; Figure 4—figure supplement 2C), further confirming that Daple serves as in intermediate protein that couples FZD7R to Gαi3. Although the interaction between ligand-activated FZD7R and Daple does not require the GBA motif (Figure 4—figure supplement 2B), the recruitment of Gαi into the complex requires a functionally intact GBA to trigger the formation of FZD7R(active)-Daple-Gαi complexes. Thus, two non-overlapping modules in-tandem within Daple's C-terminus cooperate to facilitate the assembly of FZD7R(active)-Daple-Gαi ternary complexes (Figure 4L)—(1) a GBA motif that binds Gαi and (2) a stretch of C-terminus (aa 1681–2024) is essential for binding to FZD7R.

### Daple competes with Dvl for binding to FZDRs and antagonizes Wnt signaling via the β-catenin/TCF/LEF pathway

**Figure 5.**
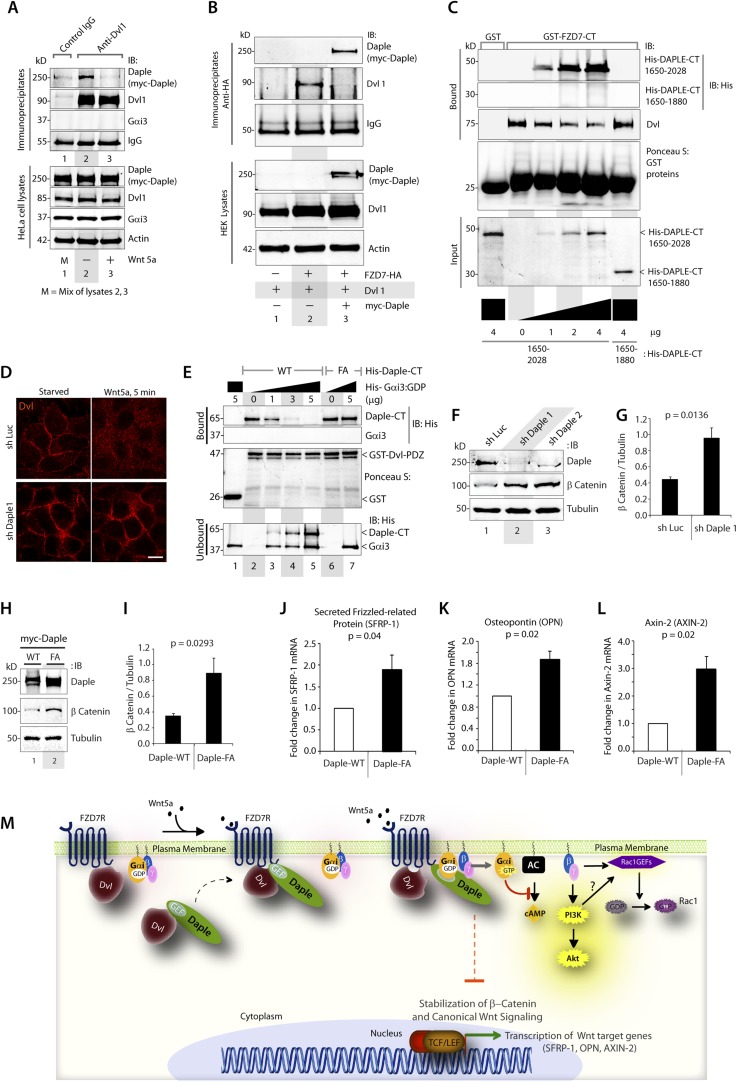
Daple competes with Dvl for binding to FZD7R and inhibits the canonical β-catenin/TCF/LEF signaling pathway via the GBA motif. (**A**) Dishevelled (Dvl)–Daple complexes are disrupted upon Wnt5a stimulation. HeLa cells cotransfected with myc-Daple-WT and Dvl were incubated in a 0.2% serum media overnight, and treated (+) or not (−) with 0.1 mg/ml Wnt5a for 5 min prior to lysis. Equal aliquots of lysates (bottom) were incubated in the presence of anti-Dvl mAb and subsequently with protein G beads. Immune complexes (top) were analyzed for Daple (myc), Dvl, and Gαi3 by IB. (**B**) Dvl and Daple compete for recruitment to FZD7 receptor in cells. Equal aliquots of lysates of HEK293 cells cotransfected with FZD7-HA with Dvl and/or myc-Daple-WT were incubated with anti-HA mAb and subsequently with protein G beads. Immune complexes were analyzed for Daple and Dvl by IB. (**C**) Daple can displace Dvl bound to the cytoplasmic tail of FZD7R in vitro. Dvl expressed in HEK cells was pre-bound to GST or GST-FZD7CT and subsequently incubated with increasing amounts of recombinant His-Daple-CT proteins as indicated. Bound proteins were analyzed for Daple (His) and Dvl by IB. (**D**) Daple is required for the ligand-stimulated dissociation of Dvl from the PM. Control (sh Luc) and Daple-depleted (sh Daple 1) Hela cells coexpressing Dvl and FZD7R were starved and stimulated with Wnt5a prior to fixation as in 4B. Fixed cells were stained for Dvl (red) and analyzed by confocal microsocpy. Bar = 10 µM. (**E**) Gαi competes with Dvl for binding to Daple in vitro. Equal aliquots of GST or GST-Dvl-PDZ (immobilized on glutathione beads) and Daple-CT (WT or FA) recombinant proteins were incubated with increasing amounts of purified His-Gαi3 as indicated. Bound (top) and unbound (supernatant; lower) proteins were analyzed for Daple-CT and Gαi3 (His) by IB. GST and GST-Dvl-PDZ were visualized by ponceau staining. (**F**) Depletion of Daple increases the levels of β-catenin. Whole-cell lysates of control (shLuc) and Daple-depleted (shDaple1 and 2) HeLa cells were analyzed for β-catenin by IB. (**G**) Bar graphs display quantification of β-catenin in **F**. Error bars represent mean ± S.D of three independent experiments. (**H**) Daple's GBA motif is required for suppression of β-catenin expression/stability. Whole-cell lysates from HeLa cells transfected with myc-Daple-WT or FA were analyzed for β-catenin expression by IB. Two biological replicates are shown. (**I**) Bar graphs display quantification of β-catenin in **H**. Error bars represent mean ± S.D of three independent experiments. (**J**, **K**, **L**) Daple's GBA motif is required for suppression of Wnt target genes. HeLa cells transfected with myc-Daple-WT or FA were analyzed for SFRP-1, OPN, AXIN-2 mRNA by qPCR. Results were normalized internally to mRNA levels of the housekeeping gene, GAPDH. Bar graphs display the fold change in each RNA (y axis) in cells expressing Daple-FA normalized to the expression in cells expressing Daple-WT. Error bars represent mean ± S.D of three independent experiments. (**M**) Schematic of working model. (From left to right) In the absence of Wnt5a ligand, Dvl remains at the PM complexed to inactive FZD7Rs, whereas Daple remains in the cytosol in complex with cytosolic Dvl, and Gαi/βγ trimers at the PM are largely inactive. Upon ligand stimulation, Dvl-Daple complexes dissociate and Daple is recruited to the cytoplasmic tails of activated receptors, Dvl is displaced from the receptor tail by Daple, Daple favors the assembly of receptor-Gαi complexes and triggers the activation of Gαi within these complexes. Activated Gαi and Gβγ subunits trigger signaling via their respective downstream intermediates (Rac1, PI3K, and cAMP). Another major consequence of these signaling events is suppression of the canonical β-catenin/TCF/LEF signaling pathway, which regulates the transcription of Wnt target genes. **DOI:** http://dx.doi.org/10.7554/eLife.07091.011

Previous studies have demonstrated that Dvl, a key scaffold protein in the Wnt signaling pathway, interacts with both FZDRs (Schulte and Bryja, 2007) and Daple (Oshita et al., 2003) and shapes both canonical and non-canonical Wnt signals. Furthermore, Dvl interferes with the engagement of Gi proteins with ligand-activated FZDRs (Kilander et al., 2014), suggesting a possible interplay between Dvl and the FZDR-Daple-Gi signaling axis we define here. First, we investigated how the ligand-dependent Daple-FZD7R interaction affects Dvl's ability to bind Daple. We found that Daple co-immunoprecipitated with Dvl exclusively in starved cells and that such complexes were undetectable after stimulation with Wnt5a (Figure 5A), indicating that the dissociation of Daple-Dvl complexes coincides with the assembly of Daple-FZD7R complexes we observed in Figure 4A. Next, we investigated how Daple affects the interaction between Dvl and FZDR. We found that expression of Daple in HEK293 cells reduces Dvl association with FZD7R in pulldown (Figure 5—figure supplement 1A) and co-IP experiments (Figure 5B), suggesting that Daple and Dvl may compete with each other for binding to FZD7R. Furthermore, immunofluorescence studies confirmed that localization of Dvl at the PM in cells expressing FZD7R was reduced within 5 min after Wnt5a stimulation (Figure 5—figure supplement 1B), which coincides with the ligand-dependent recruitment of Daple (Figure 4B). We found that Daple and Dvl actually compete for binding to FZD7R because increasing amounts of purified His-Daple-CT (1650–2028), but not a shorter fragment (His-Daple 1650–1880, which lacks the FZD7R-binding region) increased the formation of Daple-FZDR complexes and reduced DvlFZD7R complexes (Figure 5C). Furthermore, immunofluorescence studies revealed, that in cells without Daple, stimulation with Wnt5a does not trigger the loss of Dvl from the PM observed in control cells (Figure 5D), suggesting that the competition we observe in vitro (Figure 5C) may occur also in cells. Taken together, these results indicate that Daple determines the relocalization of Dvl upon Wnt5a stimulation by displacing the latter from FZDRs.

**Figure 5—figure supplement 1.**
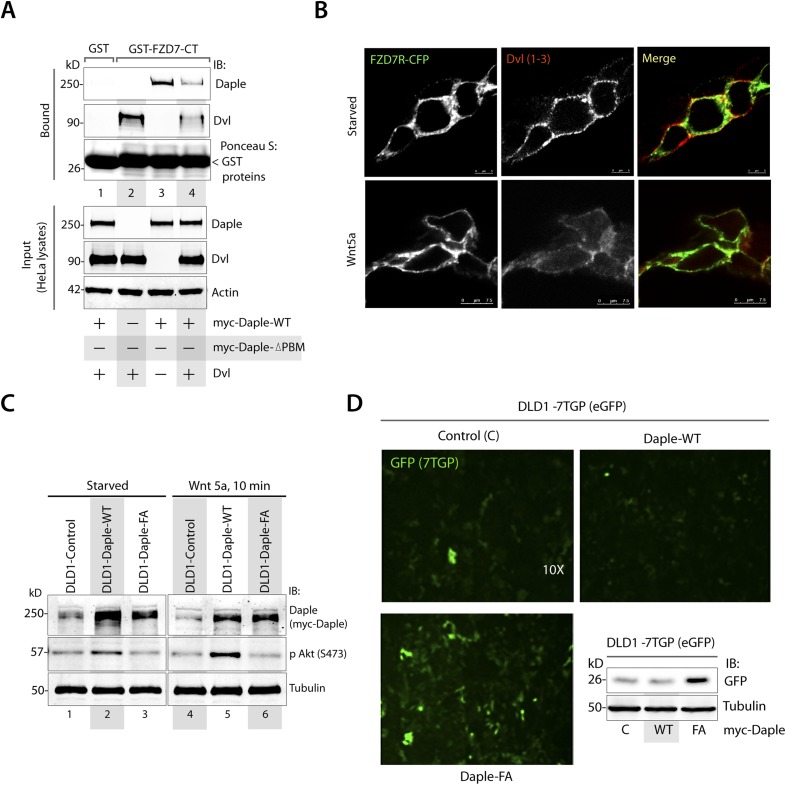
Daple competes with Dvl for binding to FZD7R and inhibits the canonical β-caenin/TCF/LEF signaling pathway. (**A**) Equal aliquots of lysates from Cos7 cells expressing Dvl1 alone (lane 2), myc-Daple alone (lane 3), or coexpressing both (lanes 1, 4) were used as source of Daple and Dvl in GST pulldown assays with recombinant, immobilized GST or GST-FZD7-CT. Bound proteins were analyzed for Dvl1 and Daple by immunoblotting (IB). Binding of each protein was higher when expressed alone (lanes 2, 3) than when co-expressed (lane 4). (**B**) Dvl loses colocalization with FZD7R at the PM after Wnt5a stimulation. HEK293 cells expressing FZD7-CFP were grown on cover slips coated with Poly-D-Lysine, starved overnight, and treated with 0.1 mg/ml Wnt5a as in 4B. Cells were fixed and stained for endogenous Dvl (red) and analyzed by confocal microscopy. (**C**, **D**) Generation and characterization of DLD1 7TGP cell lines stably expressing Daple. (**C**) DLD1 7TGP cell lines stably expressing Daple-WT or FA were starved and stimulated analyzed for Daple expression and phosphorylation of Akt by immunoblotting (IB). (**D**) Images display representative fields from monolayers of DLD1 cells grown in 0.2% FBS by fluorescence microscopy. The intensity of eGFP signals denotes Wnt transcriptional activity. Inset shows immunoblots (IB) of equal aliquots of whole-cell lysates of DLD1-7TGP cells expressing control vector, Daple-WT, or Daple-FA. Compared to DLD1 cells expressing Daple-WT, those expressing Daple-FA also express higher levels of GFP protein, indicative of higher Wnt transcriptional activity. **DOI:** http://dx.doi.org/10.7554/eLife.07091.012

**Figure 5—figure supplement 2.**
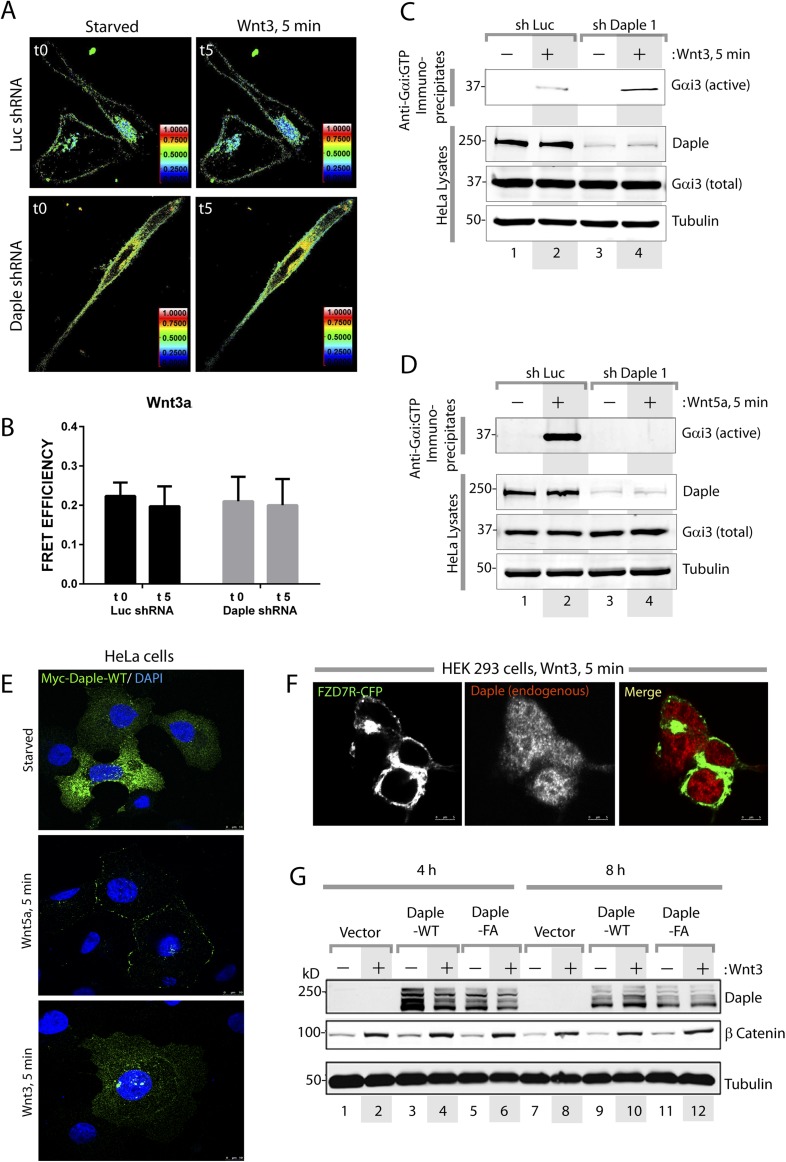
Daple and its GBA motif do not affect canonical Wnt signaling. (**A**–**D**) Daple does not activate Gi after Wnt3 stimulation. (**A**, **B**) Control (Luc shRNA) or Daple-depleted (Daple shRNA) HeLa cells were cotransfected with Gαi1-YFP, Gβ-CFP, and untagged Gγ, serum starved overnight (0.2% FBS) and subsequently stimulated with either Wnt3 and analyzed for FRET by confocal microscopy. Representative freeze-frame images from live-cell movies are shown (**A**), which display intensities of acceptor emission due to FRET in each pixel. Activation of Gi was insignificant, as determined by continued interaction (i.e., continued FRET) between Gαi1 and Gβ1γ2 both before and after Wnt3 stimulation (compare t0 and t5) both in control (Luc shRNA) and in Daple-depleted HeLa cells. Bar graphs (**B**) display FRET intensities observed in control (Luc shRNA) vs Daple-depleted HeLa cells. Error bars representing mean ± S.D. of 5 randomly chosen ROIs at the PM per cell, from 2 to 3 cells per experiment, from three independent experiments. These results are in striking contrast to the findings after Wnt5a stimulation (see Figure 2I–L in manuscript). (**C**, **D**) Control (Luc shRNA) or Daple-depleted (Daple shRNA) HeLa cells were serum-starved (0.2% FBS) and treated (+) or not (−) with Wnt3 (**C**) or Wnt5a (**D**) for 15 min prior to lysis. Equal aliquots of lists were subjected to IP with antibodies that selectively recognize active Gαi subunits in their GTP-bound state. Immune complexes (top) and lysates (bottom) were analyzed for active Gαi3:GTP and total Gαi3 by IB. Wnt5a robustly activates Gαi3, and this activation is abolished upon Daple depletion, whereas Wnt3 marginally activates Gαi3 and this activation is not diminished upon Daple depletion. (**E**) Myc-Daple is translocated to the PM after Wnt5a, but not after Wnt3 stimulation. HeLa cells were transfected with myc-tagged Daple-WT, serum starved overnight (0.2% FBS) and subsequently stimulated with either Wnt5a or Wnt3 as indicated. Cells were fixed at 5 min after ligand stimulation and analyzed for localization of myc-Daple (Green) by immunofluorescence. Myc-Daple was found in cytosolic distribution prior to ligand stimulation in starved cells. Upon stimulation with Wnt5a Daple was found to localize sharply at the PM. Upon stimulation with Wnt3 myc-Daple remained in cytosolic location. (**F**) Endogenous Daple is translocated to the PM after Wnt5a, but not after Wnt3 stimulation. HEK cells transfected with CFP-tagged FZD7R were serum starved for 24 hr (0% FBS), and subsequently stimulated with either Wnt5a or Wnt3 as indicated. Cells were fixed at 5 min after ligand stimulation and analyzed for localization of endogenous Daple by immunofluorescence. Daple was found in cytosolic distribution prior to ligand stimulation in starved cells (see Figure 4B). Upon stimulation with Wnt3, Daple remained in cytosolic location, however, upon stimulation with Wnt5a Daple was found to localize at the PM, where it colocalized with FZD7R (see Figure 4B). (**G**) Daple's GBA motif does not affect Wnt3-dependent stabilization of β Catenin. HEK293 cells were transfected with Daple-WT or FA mutant, serum starved (0% FBS) for 24 hr, and subsequently stimulated with Wnt3 for 4 hr (lanes 1–6), 8 hr (lanes 7–12), or 20 hr (lanes 13–18) prior to lysis. Equal aliquots of cytoplasmic extracts were analyzed for β Catenin, Daple, and tubulin by IB. β Catenin was stabilized (increased, compare even lanes with odd lanes) in each condition tested, without significant differences between Daple-WT vs Daple-FA at any time points observed. A representative experiment from a total of four independent experiments is shown. **DOI:** http://dx.doi.org/10.7554/eLife.07091.013

Because the interplay between of Daple and Dvl is modulated by Wnt5a and the GBA motif of Daple regulates Wnt5a-signaling responses, next, we examined if/how the Daple-Gαi interaction affects the interaction between Dvl and Daple. In in vitro competition assays with recombinant proteins, we found that binding between Daple and Dvl was reduced with increasing amounts of His-Gαi3 (Figure 5E). No such reduction was noted when the Daple-CT-WT was replaced by the GBA-deficient FA mutant (that cannot bind G proteins) in the above assays. These findings indicate that Gαi3 competes with Dvl for binding to Daple-CT, and that an intact GBA motif is essential for such competition. Together, these results suggest that the Gαi-Daple and Daple-FZD7R interactions we describe here have at least two major effects on the interplay between Daple, Dvl, and FZD7R: (1) Daple and Dvl compete for binding to the C-terminus of FZD7R and (2) Gαi and Dvl compete for binding to the C-terminus of Daple. Consequently, stimulation with Wnt5a triggers the dissociation of Daple-Dvl and FZD7R-Dvl complexes and favors the assembly of FZD7R-Daple-Gαi signaling complexes at the PM in detriment of FZD7R-Dvl complexes.

Next, we asked what might be the consequences of replacing Dvl with Daple and activation of G proteins in the vicinity of ligand-activated FZD7R on β-catenin/TCF/LEF signaling. Prior studies have demonstrated that activation of G proteins downstream of FZDRs is sufficient for antagonistic suppression of β-catenin-dependent signaling (Slusarski et al., 1997a, 1997b). Others have implicated binding of Dvl to FZDRs is required for the enhancement of the β-catenin/TCF/LEF pathway of signaling (Gao and Chen, 2010). We asked if activation of G proteins via Daple's GBA motif may antagonize β-catenin stability/signaling. We found that HeLa cells without Daple (Figure 5F,G) or those expressing the GEF-deficient Daple FA mutant (Figure 5H,I) had increased levels of β-catenin protein compared to respective controls, indicating that Daple and its GBA motif are required for maintenance of low levels of β-catenin, and that in their absence β-catenin is stabilized. Consistently, increased stability of β-catenin was also associated with enhanced transcription of downstream target genes SFRP-1, Osteopontin, and Axin-2 (Figure 5J–L). Similar results were obtained when we analyzed the β-catenin/TCF/LEF pathway in DLD1 colon cancer cells stably expressing Daple-WT or FA mutant (Figure 5—figure supplement 1C) using 7-TGP, an eGFP expressing Wnt activity reporter construct (Fuerer and Nusse, 2010). Wnt activity was enhanced in cells expressing Daple-FA, but not Daple-WT (Figure 5—figure supplement 1D), consistent with our prior findings in HeLa cells. Finally, we found that Daple specifically functions within the non-canonical Wnt signaling cascade and not within the canonical Wnt pathway, for example, stimulation of the canonical Wnt pathway with Wnt3a did not require Daple to activate Gi (Figure 5—figure supplement 2A–D), did not trigger the recruitment of Daple to the PM (Figure 5—figure supplement 2E,F), and did not affect the stabilization of β-catenin (Figure 5—figure supplement 2G). These results suggest that the repressive effects of Daple we observe on the β-catenin/TCF/LEF pathway (Figure 5J–L) are likely due to enhancement of the antagonistic non-canonical Wnt signaling pathway.

Taken together, these results support an overall model (Figure 5M) in which Daple orchestrates non-canonical Wnt signaling by favoring the recruitment and activation of G proteins and displacement of Dvl from activated FZDRs upon Wnt5a stimulation. This leads to enhancement of Akt and Rac1 signaling (via ‘free’ Gβγ) and suppression of cellular cAMP (via Gαi:GTP), which is accompanied by diminished activity of the β-catenin/TCF/LEF pathway.

### The GBA motif in Daple triggers tumor cell migration but suppresses growth and proliferation

**Figure 6.**
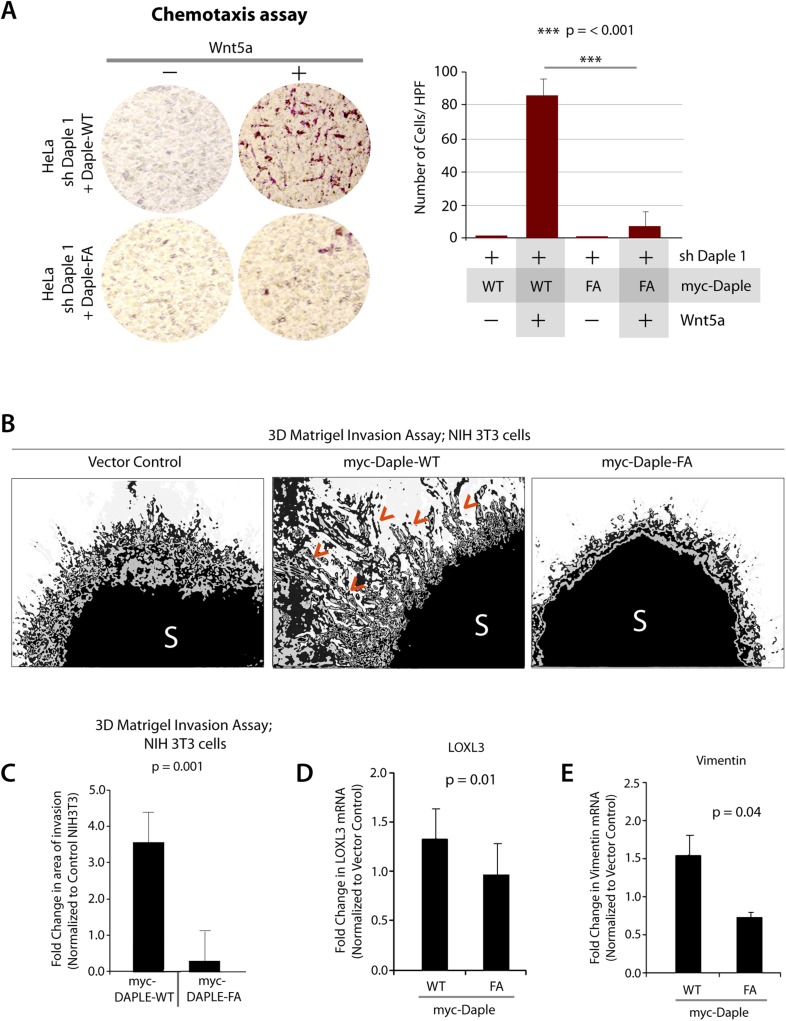
Daple enhances cell migration and invasion via its GBA motif. (**A**) Daple WT, but not FA triggers chemotactic migration towards Wnt5a. Daple-depleted HeLa cells (sh Daple 1) stably expressing Daple-WT or Daple-FA were analyzed for their ability to migrate towards Wnt5a (+) or vehicle control (−) in transwell assays. Cells were allowed to migrate for 24 hr, fixed and stained with Giemsa. The number of migrating cells was averaged from 20 field-of view images per experiment. Data are presented as mean ± SEM; n = 3. HPF = high-power field. Lysates of cells used in this assay were analyzed for Daple expression by IB (see Figure 6—figure supplement 1C). (**B**, **C**) Daple WT, but not FA triggers cell invasion. Spheroids (S) of NIH3T3 cells expressing vector control, myc-Daple-WT, or FA were analyzed for their ability to invade matrigel in response to serum stimulation using a Cultrex-3D Spheroid Invasion Kit (Trevigen). An increase of invading cells (arrowheads; **B**) were noted only from the edge of tumor spheroids formed by cells expressing myc-Daple-WT, but not FA. Area of invasion was quantified using ImageJ (as shown with interrupted blue line in Figure 6—figure supplement 1D). (**C**) Bar graphs display area of invasion observed in Daple WT and Daple FA expressing cells. Error bars representing mean ± S.D of three independent experiments. (**D**, **E**) Daple-WT, but not Daple-FA enhances the expression of genes that trigger epithelial–mesenchymal transition (EMT). mRNA expression of the EMT markers, LOXL3, and Vimentin were analyzed by qPCR. Results were normalized internally to mRNA levels of the housekeeping gene, GAPDH. Bar graphs display the fold change in each RNA (y axis) normalized to the expression in cells expressing vector control. Error bars represent mean ± S.E.M of three independent experiments. **DOI:** http://dx.doi.org/10.7554/eLife.07091.014

Next, we investigated how non-canonical Wnt signaling via the Wnt5a/FZDR-Daple-Gαi axis impacts cancer cell behavior. We first analyzed the cellular phenotypes that are modulated by Wnt5a and non-canonical Wnt signaling during different stages of cancer progression (McDonald and Silver, 2009). In the normal mucosa, this pathway serves as a tumor-suppressor, by antagonizing the canonical Wnt-β-catenin signaling pathway (Torres et al., 1996; MacLeod et al., 2007; Ying et al., 2007, 2008; Chien et al., 2009), whereas in advanced tumors it triggers cell migration/invasion by enhancing PI3K-Akt and Rac1 pathways and the formation of actin stress fibers (Nishita et al., 2010; Liu et al., 2013; Zhang et al., 2014). Consistent with the role of Daple's GBA motif in enhancement of Akt and Rac1 activities (Figure 3), we found that monolayers of Daple-depleted HeLa cells stably expressing Daple-WT, but not Daple FA efficiently closed wounds and generated actin stress fibers (Figure 6—figure supplement 1A–C) and migrated efficiently along a gradient of Wnt5a in chemotaxis assays (Figure 6A). To determine if Daple can trigger cell invasion through basement membrane proteins, we carried out 3-D matrigel invasion assays. Non-invasive NIH3T3 cells (Albini et al., 1987) stably expressing Daple-WT, Daple-FA, or vector control were grown into tumor spheroids and subsequently analyzed for cell invasion through matrix (Figure 6B,C). Enhanced invasion (as determined by the area of invasion; Figure 6—figure supplement 1B) was detected exclusively in the presence of Daple-WT, but not in cells expressing control vector or Daple-FA, indicating that Daple is sufficient to trigger cell invasion, and that a functionally intact GBA motif is essential. Compared to cells expressing Daple-FA, those expressing Daple-WT had significantly higher expression of Lox-L3 and Vimentin, two genes commonly associated with epithelial–mesenchymal transition (EMT) (Figure 6D,E), indicating that higher invasiveness was accompanied by an EMT gene signature.

**Figure 6—figure supplement 1.**
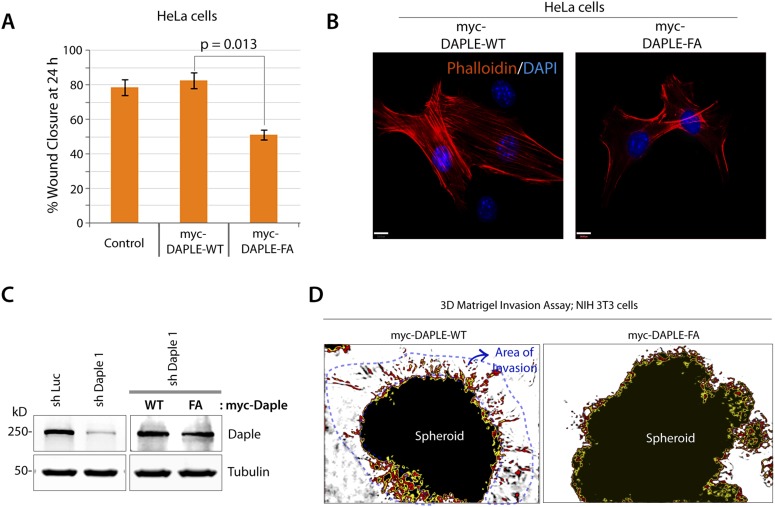
Daple enhances cell migration, promotes formation of actin stress-fibers, and triggers invasion, all via its GBA motif. (**A**) Daple-FA, but not Daple-WT inhibits 2-D cell migration. Confluent monolayers of HeLa cells transiently transfected with myc-Daple WT or FA (∼90% efficacy of transfection confirmed by immunofluorescence) or control vector were scratch-wounded and incubated for 24 hr in a 0.2% serum media. Wound closure was monitored and quantified as detailed in ‘Materials and methods’. % wound closure (y axis) in various cell lines are displayed as bar graphs. For each cell line, ∼3–5 scratch-wounds were analyzed in each assay. Expression of Daple-FA significantly delays wound closure. Error bars represent mean ± S.E.M of three independent experiments. (**B**) Daple-WT, but not Daple-FA triggers formation of actin stress fibers. Daple-depleted HeLa cells transiently transfected with myc-Daple WT or FA were grown on cover slips in the presence of 0.2% FBS, fixed, and subsequently analyzed for actin cytoskeleton patterns by staining with Phalloidin (red). Abundance of stress fibers running across the cell bodies was seen in cells expressing Daple-WT. Blue = DAPI/nucleus. Bars = 10 µm. (**C**) Whole-cell lysates of HeLa cell lines used in transwell chemotaxis assays in Figure 6A were analyzed for Daple expression by IB. (**D**) Daple WT, but not FA triggers cell invasion. Spheroids of NIH3T3 cells expressing myc-Daple WT and FA were analyzed for their ability to invade matrigel in response to serum stimulation using a Cultrex-3D Spheroid Invasion Kit (Trevigen; see ‘Materials and methods’). Tracks created by invading cells were noted only in cells expressing myc-Daple WT. Area of invasion was quantified using ImageJ (as shown with interrupted blue line). Bar graphs showing the quantification of the area of invasion are shown in Figure 6C. **DOI:** http://dx.doi.org/10.7554/eLife.07091.015

**Figure 7.**
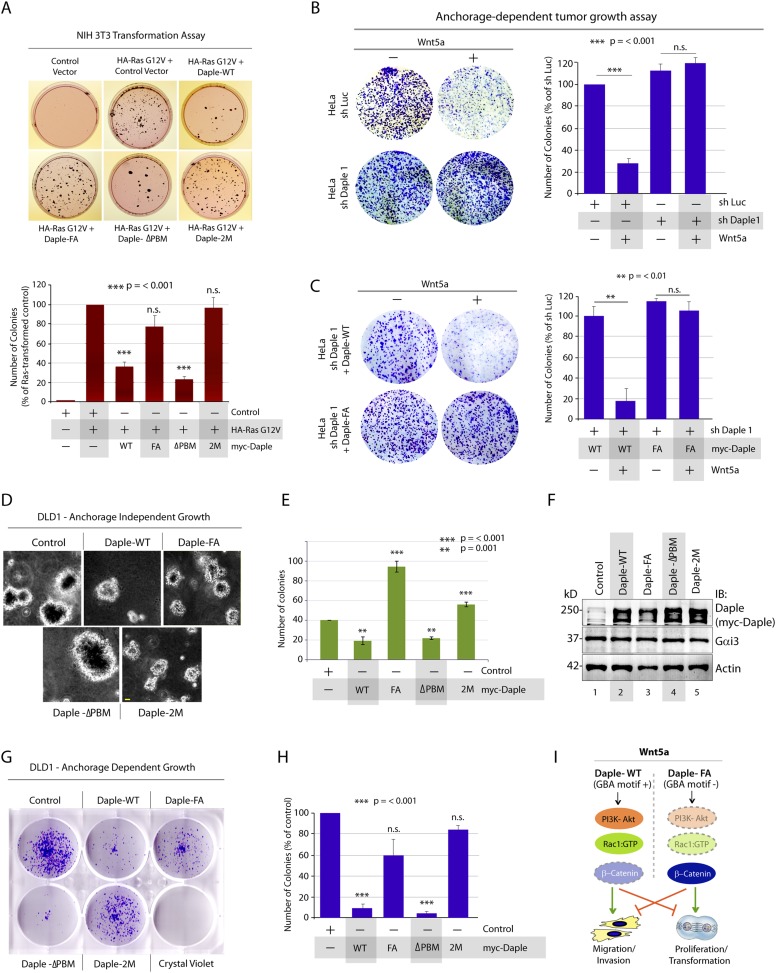
Daple suppresses proliferation and tumorigenesis via its GBA motif. (**A**) Daple's GBA motif is required for inhibition of cell transformation induced by oncogenic KRas. NIH3T3 cells stably expressing HA-KRas G12V alone or coexpressing HA-KRas G12V with myc-Daple-WT or various mutants were analyzed for their ability to form colonies in soft agar prior to staining with MTT. The top panel displays representative images of colony-containing plates. Bar graphs in the lower panel shows % inhibition of colony formation (y axis) by each Daple construct compared to NIH3T3 cells transformed with KRas G12V alone. Lysates of NIH3T3 cells were analyzed for Daple and Ras constructs by IB (see Figure 7—figure supplement 1B). (**B**) Daple is required for inhibition of anchorage-dependent tumor growth by Wnt5a. Control (shLuc) and Daple-depleted (sh Daple 1) HeLa cells were analyzed for their ability to form colonies on plastic plates in the presence (+) or absence (−) of Wnt5a during a 2-week period prior to fixation and staining with crystal violet. Left panel shows the photograph of the crystal violet-stained wells of a 6-well plate. The number of colonies was counted by ImageJ (Colony counter). Right panel shows bar graphs that display the % inhibition of colony formation (y axis) seen in each condition normalized to control (shLuc) HeLa cells. (**C**) Daple's GBA motif is required for inhibition of anchorage-dependent tumor growth by Wnt5a. Daple-depleted (sh Daple 1) HeLa cells stably expressing either Daple WT or FA were analyzed for their ability to form colonies on plastic plates in the presence (+) or absence (−) of Wnt5a prior to fixation and staining with crystal violet, photographed and analyzed as in **B**. Left panel shows the photograph of the crystal violet-stained wells of a 6-well plate. Right panel shows bar graphs that display the % inhibition of colony formation (y axis) seen in each condition normalized to control (shLuc) HeLa cells. (**D**–**F**) Daple's GBA motif is required for inhibition of anchorage-independent tumor growth. DLD1 cells expressing either control vector or various myc-Daple constructs were analyzed for their ability to form colonies in soft agar for 2–3 weeks. In panel **D**, representative fields photographed at 20× magnification are shown. The number of colonies was counted by light microscopy throughout the depth of the matrix in 15 randomly chosen fields. In panel **E**, bar graphs display the number of colonies (y axis) seen in each cell line in **D**. In panel **F**, lysates of DLD1 cells used in **D** were analyzed for Daple constructs by IB. (**G**, **H**) Daple's GBA motif is required for inhibition of anchorage-dependent tumor growth. DLD1 cells used in **D** were analyzed for their ability to form adherent colonies on plastic plates during 2–3 weeks prior to fixation and staining with crystal violet. In panel **G**, photograph of the crystal violet-stained 6-well plate is displayed. The number of colonies was counted by ImageJ (Colony counter). In panel **H**, bar graphs display the % inhibition of colony formation (y axis) seen in each cell line in G normalized to control DLD1 cells. (**I**) Schematic summary. Modulation of G protein activity by Daple's GBA motif is a key determinant of cellular phenotype(s) triggered by Wnt5a. In cells expressing Daple-WT, a functionally intact GBA motif (+) can activate Gαi, enhance PM-based motogenic signals (PI3K-Akt and Rac1 activation), trigger EMT and cell migration/invasion. In cells expression Daple-FA, without the functional GBA motif (−) G protein remains inactive, non-canonical Wnt signaling is suppressed, which increases stability of β-catenin and upregulation of Wnt target genes, resulting in increased transformation, proliferation, and tumor cell growth. **DOI:** http://dx.doi.org/10.7554/eLife.07091.016

Next, we investigated the role of Daple and its GBA motif in the modulation of other key cellular phenotypes regulated by non-canonical Wnt signaling during tumorigenesis, that is, cell proliferation, transformation, and growth (Niehrs and Acebron, 2012; Jamieson et al., 2014). For this, we used three cell lines: HeLa cell lines, the constitutively active Ras-transformed NIH3T3 cells, and the DLD1 colorectal cancer cells in which transformation is driven by hyperactive β-catenin signaling in addition to active Ras mutations. We chose to study DLD1 colorectal cancer cells because Daple is virtually undetectable in these cells compared to normal colon (data not shown), thereby allowing us to reconstitute expression exogenously and analyze the effect of WT and mutant Daple constructs without significant interference due to the endogenous protein. Expression of Daple-WT reduced the number of colonies formed by Ras-transformed NIH3T3 in soft-agar by ∼65% (Figure 7A; Figure 7—figure supplement 1A), indicating that Daple's GBA motif is required for suppressing neoplastic transformation. The mitotic index, as determined by the presence of phosphorylated Histone H3 in the nucleus (Hans and Dimitrov, 2001), was higher in HeLa cells expressing Daple-FA compared to those expressing Daple-WT (Figure 7—figure supplement 1B), indicating that Daple's GBA motif suppresses mitosis. When we assessed the tumor-suppressive effect of Wnt5a on HeLa cells in anchorage-dependent tumor growth assays, we found that tumor growth was suppressed in the control cells, but such suppression was lost in cells depleted of endogenous Daple (Figure 7B). This loss of tumor-suppressive effect of Wnt5a was restored by expressing Daple-WT but not by expressing the Daple-FA mutant (Figure 7C), indicating that a functionally intact GBA motif in Daple is essential for Wnt5a to exert its tumor suppressive effects. Daple-WT also inhibited anchorage-independent tumor growth of DLD1 cells by ∼50% (Figure 7D–F), and inhibited anchorage-dependent tumor growth of DLD1 cells by ∼90% (Figure 7G,H), demonstrating that Daple suppresses cellular transformation and growth across all assays. This tumor suppressive effect was mediated via the GBA motif because, compared to Daple-WT, expression of Daple-FA not only failed to inhibit cell transformation (Figure 7A) and growth (Figure 7C,H) but also enhanced oncogenicity (Figure 7E). Noteworthy, expression of a Daple mutant that cannot bind Dvl (Daple-ΔPBM) but has an intact GBA motif retained the tumor suppressive properties of Daple-WT across all assays, whereas a mutant that lacks both the GBA and the Dvl-binding PBM motifs (Daple-2M) mirrored the phenotype of the FA mutant, indicating that the G protein regulatory GBA motif, and not the Dvl-binding PBM motif is essential for the tumor suppressive function of Daple. Taken together, these findings demonstrate that Daple inhibits cell transformation and proliferation during tumor growth, but enhances cell motility and cytoskeletal remodeling during invasion; both require the GBA motif, which regulates G protein activity (Figure 7I).

**Figure 7—figure supplement 1.**
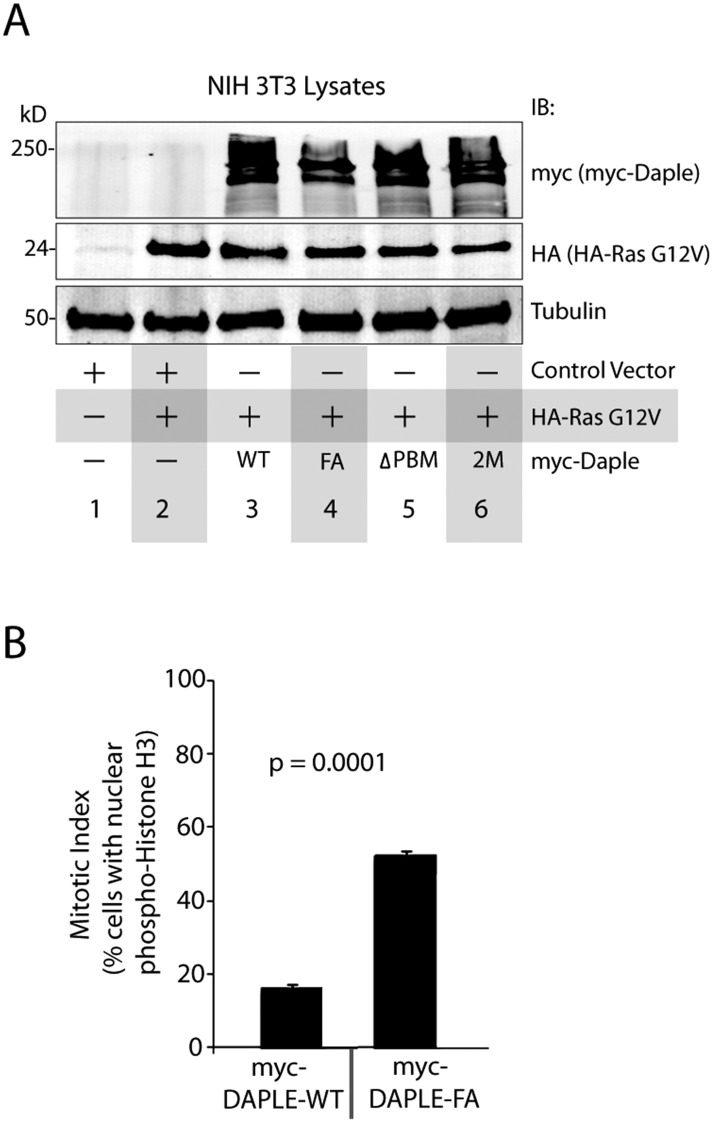
Daple suppresses cell proliferation via its GBA motif. (**A**) Compared to cells expressing Daple-WT, those expressing Daple-FA have higher mitotic index, as determined by nuclear localization of phosphorylated histone H3. HeLa cells expressing myc-Daple WT or FA were grown on cover slips in the presence of 0.2% FBS, fixed, and stained for phospho-histone H3 and DAPI. Bar graphs display % cells with nuclear phospho-histone H3 (y axis). Error bars representing mean ± S.D. of three independent experiments. (**B**) Lysates of NIH3T3 cells NIH3T3 cells used in Ras-induced transformation assays (see Figure 7A) were analyzed for Daple and Ras constructs by IB. **DOI:** http://dx.doi.org/10.7554/eLife.07091.017

### Expression of Daple is dysregulated during cancer progression

**Figure 8.**
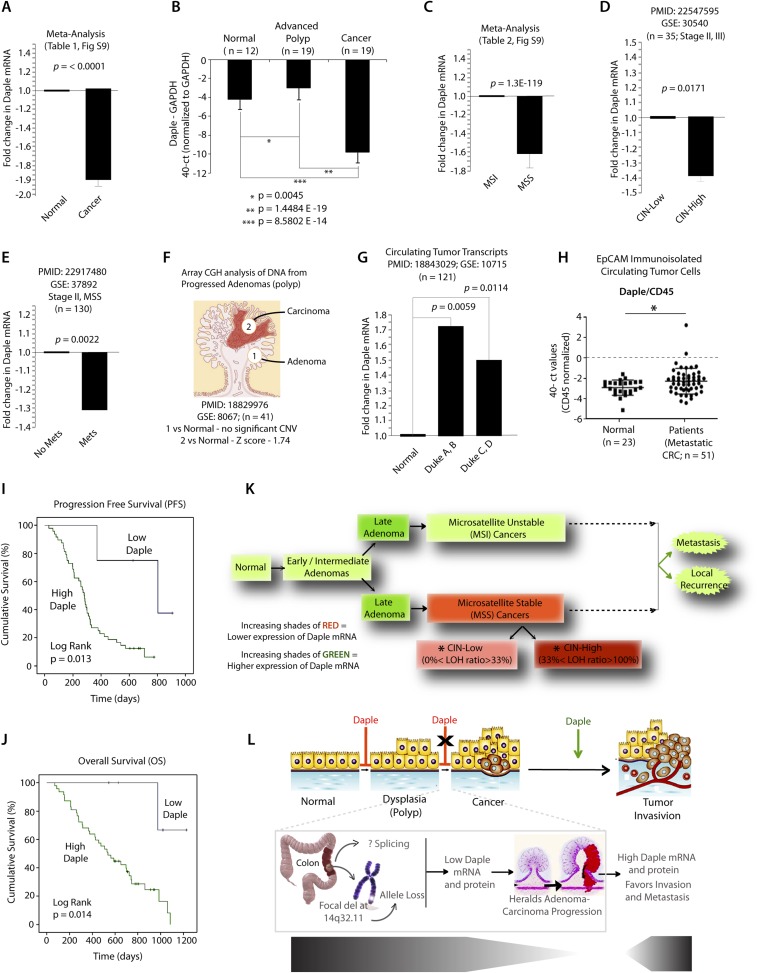
Expression of Daple mRNA is suppressed during oncogenesis by copy number loss, but expressed later during metastasis. (**A**) Daple mRNA is downregulated in colorectal cancers. A meta-analysis was performed using all the available high-throughput microarray data from *Genomic Spatial Event* (GSE) *database* (see Figure 8—source data 1) to compare the levels of expression of Daple mRNA in colorectal cancer vs matched normal controls. Bar graphs display the results of such meta-analysis as fold change in Daple mRNA (y axis) in colorectal carcinomas normalized to matched normal controls. (**B**) Daple mRNA is downregulated during the adenoma-to-carcinoma step of oncogenesis in the colon. Daple mRNA was analyzed by qPCR in normal colon, advanced adenomas, and colorectal carcinomas. Bar graphs display the relative levels of Daple mRNA normalized to GAPDH, as determined by the calculation 2 − ΔCT with reference to an absolute baseline CT of 40 cycles. Error bars represent mean ± S.D. (**C**) Daple mRNA is downregulated in microsatellite stable (MSS), but not microsatellite unstable (MSI) colorectal cancers. A meta-analysis was performed using all the available high-throughput microarray data from GSE *database* (see Figure 8—source data 2) to compare the levels of expression of Daple mRNA in MSI vs MSS colorectal cancers vs their respective matched normal controls. Bar graphs display the results of such meta-analysis as fold change in Daple mRNA (y axis) in colorectal carcinomas normalized to normal controls. (**D**) Downregulation in Daple mRNA in MSS colorectal cancers directly correlates with the degree of chromosomal instability (CIN) in the tumor. High-throughput microarray data from GSE *database* (PMID: 22547595, GSE: 30,540) were analyzed for the levels of expression of Daple mRNA in MSS colorectal cancers (stages II and III) with varying degrees of CIN [CIN-low (LOH ratio <33%) and CIN-high (LOH ratio ≥33%)] and compared to MSI tumors. Bar graphs display the results of such analysis as fold change in Daple mRNA (y axis) in CIN-low or CIN-high colorectal carcinomas compared to MSI tumors. (**E**) Downregulation of Daple mRNA in the primary tumor early during cancer progression prognosticates tumor recurrence/metastasis. High-throughput microarray data from GSE *database* (PMID: 22917480, GSE: 37,892) were analyzed for the levels of expression of Daple mRNA in 130 stage II MSS tumors without (No Mets) or with (Mets) tumor recurrence/metastatic progression. (**F**) Loss of copy number for CCDC88C (DAPLE gene) occurs at the late stages of adenoma-to-carcinoma progression. Array comparative genomic hybridization data from GSE *database* were analyzed for ccdc88c copy number variations (CNVs) in 41 progressed adenomas (i.e., adenomas that present a focus of cancer). Progressed adenomas were analyzed for CNVs relative toploidy level in the DNA in laser-microdissected adenoma and carcinoma fractions and compared to adjacent normal epithelial fractions as matched controls. (**G**) Cell-free mRNA transcripts of Daple are detected in patients with colorectal cancer, but not in normal control subjects. Microarray data from GSE *database* (PMID: 18843029, GSE: 10,715) were analyzed for Daple mRNA expression in peripheral blood samples of healthy subjects (n = 11) and of 121 patients with early (Dukes **A**, **B**) or late (Duke's **C**, **D**) stages of colorectal cancer. (**H**) Levels of Daple mRNA are frequently elevated in EpCAM (epithelial cell adhesion molecule) immunoisolated circulating tumor cells (CTCs) from patients with metastatic colorectal cancer, compared to normal subjects. Immunoisolated CTC fractions from the peripheral blood of 51 patients with metastatic (stage IV) colorectal cancer or from healthy subjects were analyzed for Daple mRNA by Taqman qPCR and adjusted for leukocyte contaminants by normalizing to CD45. Scatter-plots display the level of Daple expression in each patient within each group. A normality test confirmed that data sets in both groups were distributed normally. No significant differences were observed in the CD45 levels between two groups (*not shown*). (**I**, **J**) High levels of Daple mRNA expression in CTCs are associated with poorer progression-free (PFS; **I**) and overall (OS; **J**) survival in patients with metastatic colorectal carcinoma. Optimal cut-off values for Daple mRNA expression were statistically derived (see detailed ‘Materials and methods’) to generate subgroups of patients with high- or low-expression levels. Time-dependent survival probabilities were estimated with the Kaplan–Meier method, and the log-rank test was used to compare the subgroups. (**K**) Schematic summarizing profile of Daple expression during oncogenic progression in the colon. Degree of upregulation (green) or downregulation (red) in Daple mRNA is indicated by increasing shades of each color during the normal-to-adenoma-to-carcinoma progression in the colon is shown. (**L**) Proposed model for how a bimodal dysregulation of tumor suppressor Daple, and resultant deregulation of non-canonical Wnt signaling may propel oncogenic progression in the colon. Daple's ability to modulate G proteins via its GBA motif exerts a potent tumor suppressive effect in the normal mucosa. Early during oncogenesis (top, from left to right), downregulation of Daple (marked by ‘X’) occurs at the step of adenoma to cancer conversion, in part by DNA copy loss (bottom) due to focal deletion affecting the long arm of Chr 14. Consequently, low expression of Daple mRNA and protein triggers transformation and tumor growth/progression. Later during cancer invasion, expression of Daple is triggered via unknown mechanisms, which favors (green arrow) tumor recurrence and prognosticates poor survival. **DOI:** http://dx.doi.org/10.7554/eLife.07091.018 10.7554/eLife.07091.019Figure 8—source data 1.Meta-analysis of Daple mRNA expression in colorectal cancer vs matched normal controls.The publicly available GSE database, a system to store, retrieve, and analyze all types of high-throughput microarray data was used to compare the levels of expression of Daple mRNA in colorectal cancer vs matched normal controls. From left to right, the columns indicate the GSE series ID, the PMID number for the respective source manuscripts, total samples analyzed in each study, fold change in Daple mRNA observed, and the significance (p-value) of any changes observed. A meta-analysis combining the p-values from these studies was analyzed by Fisher's method and displayed as bar graphs in Figure 8A.**DOI:** http://dx.doi.org/10.7554/eLife.07091.019 10.7554/eLife.07091.020Figure 8—source data 2.Meta-analysis of Daple mRNA expression in microsatellite unstable (MSI) vs stable (MSS) colorectal cancers.The publicly available GSE database was used to compare the levels of expression of Daple mRNA in MSI vs MSS colorectal cancers. From left to right, the columns indicate the GSE series ID, the PMID number for the respective source manuscripts, total samples analyzed in each study, fold change in Daple mRNA observed, and the significance (p-value) of any changes observed. A meta-analysis combining the p-values from these studies was analyzed by Fisher's method and displayed as bar graphs in Figure 8C.**DOI:** http://dx.doi.org/10.7554/eLife.07091.020 10.7554/eLife.07091.021Figure 8—source data 3.Daple expression in CTCs correlates with markers of EMT.Expression of Daple, ZEB2, and LOXL3 mRNA were analyzed in CTCs immunoisolated from 50 patients with metastatic colorectal cancer. An analysis of the Pearson's correlation coefficient for each pair of genes shows that higher expression of Daple is significantly associated with higher expression of ZEB2 and LOXL3, two genes implicated in triggering EMT.**DOI:** http://dx.doi.org/10.7554/eLife.07091.021

Because Wnt5a and the non-canonical Wnt pathway is known to be dysregulated during cancer progression (i.e., suppressed early during neoplastic transformation and upregulated later during metastasis) (McDonald and Silver, 2009), next, we asked whether the expression of Daple is similarly altered during oncogenesis in the colon. Analysis of several publicly available microarray databases revealed expression of Daple mRNA was reduced by ∼twofold in adenocarcinomas of the colon or rectum compared to matched normals (Figure 8A; Figure 8—figure supplement 1A,B; Figure 8—source data 1). When we analyzed Daple mRNA in another cohort of patients by quantitative PCR (qPCR), we confirmed that Daple is indeed downregulated in cancers (Figure 8B), but not in the precancerous advanced polyps (defined as any adenoma with >25% villous features, or ≥1.0 cm in size, or high-grade dysplasia); the latter showed a modest upregulation in Daple mRNA (Figure 8B). This suggests that the suppression of Daple is fairly late during oncogenesis coinciding with late adenoma-to-cancer progression. Meta-analysis of various microarray databases at The Cancer Genome Atlas (TCGA; www.cancergenome.nih.gov) further revealed that expression of Daple mRNA is significantly suppressed in microsatellite stable (MSS) colorectal tumors, which account for ∼85% of all colorectal cancers and are characterized by the presence of chromosomal instability (CIN) (Figure 8C, Figure 8—figure supplement 1C; Figure 8—source data 2), whereas tumors with high degree of microsatellite instability (MSI-high) express at levels similar to normal colon (Figure 8C; Figure 8—figure supplement 1C). Among MSS tumors, the degree of suppression of Daple correlated with the degree of CIN (Figure 8D). Furthermore, as shown previously in the case of other tumor suppressors (Pino and Chung, 2010), we found that suppression of Daple mRNA in the primary tumors at the time of diagnosis was associated with disease progression, as determined by formation of distant metastasis in a cohort of patients with stage II colorectal cancers (Figure 8E). Taken together, these results indicate that expression of Daple is frequently reduced during oncogenesis, that such reduction is more common in the setting of CIN, and that reduced expression of Daple in primary tumors may predict disease progression.

**Figure 8—figure supplement 1.**
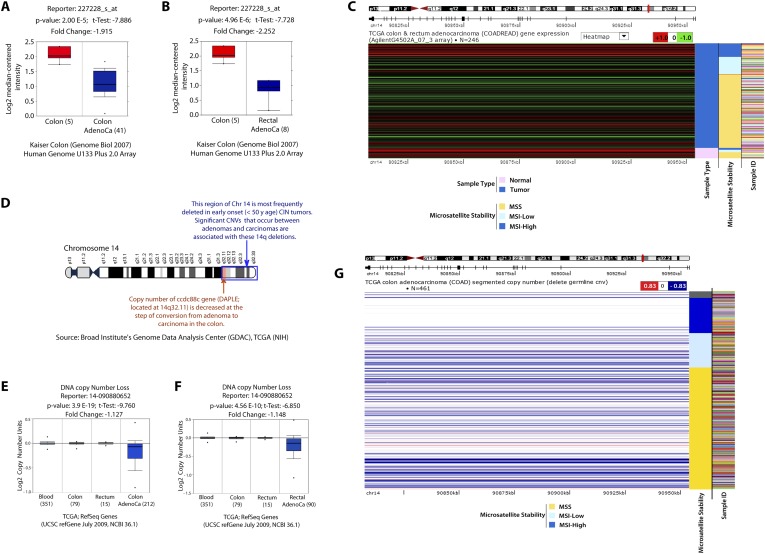
Expression of Daple mRNA is suppressed in colorectal cancers, in part by copy number loss. (**A**, **B**) Publicly available Kaiser Colon database was analyzed for Daple mRNA expression in adenocarcinomas of the colon (**A**) and rectum (**B**) and their respective normal controls. Daple mRNA expression levels are displayed using log2 median-centered ratio boxplots for normal vs cancer that were generated using the UCSC Cancer Genome Browser. Numbers in parenthesis represent total number of samples analyzed. (**C**) The TCGA colon cancer database was analyzed for Daple mRNA expression in 246 colorectal adenocarcinomas. Daple mRNA expression levels are displayed as heat maps generated using the UCSC Cancer Genome Browser. Red = High Daple; green = Low Daple. Samples are arranged by sample type (normal vs cancer) and microsatellite status (MSI low or high vs MSS) as indicated on the right margin of the heat map. (**D**) Schematic of chromosome 14 is shown. Ccdc88c gene, which encodes Daple (red arow) is located within a frequently deleted region of Chr 14 (blue box). (**E**, **F**) Publicly available TCGA database was analyzed for number of copies of Daple gene in adenocarcinomas of the colon (**E**) and rectum (**F**) compared to matched normal mucosa and in blood cells. Copy number units of ccdc88c (Daple) in various matched samples are displayed using log2 median-centered ratio boxplots for that were generated using the UCSC Cancer Genome Browser. Numbers in parenthesis represent total number of samples analyzed. Compared to matched normal mucosa or peripheral blood, lower copy numbers of Daple gene were observed in adenocarcinomas of colon and rectum. (**G**) The TCGA colon cancer database was analyzed for the relationship between Daple copy number loss and microsatellite status in 461 tumor samples. Daple copy number in each tumor is displayed as heat map (blue = loss; red = gain) generated using the UCSC Cancer Genome Browser. Samples are arranged by microsatellite status (MSI low or high vs MSS) as indicated on the right margin of the heat map. A large majority of tumors had copy number loss (blue), but not gain (red). Tumors that had a loss of copy for the Daple gene (blue) are invariably MSS tumors, or MSI-low tumors. Copy number loss is virtually absent among MSI-high tumors. **DOI:** http://dx.doi.org/10.7554/eLife.07091.022

While seeking clues into how Daple might be downregulated in some tumors, but not all, we noted that ccdc88c, the gene that encodes Daple is located in a region of Chr 14 (14q32.11) that is most frequently deleted in early onset (<50 y) colorectal tumors (Figure 8—figure supplement 1D). In fact, 14q deletions are most often associated with significant copy number variations that occur during adenoma-to-carcinoma conversion (Tsafrir et al., 2006). An analysis of microarray-based comparative genomic hybridization obtained from polyps that had progressed to cancer revealed that significant loss of Daple copy number was observed in the carcinoma portion, but not in the adenoma portion of these advanced polyps compared to matched normal tissue (Figure 8F). Loss of Daple copy number was noted in adenocarcinomas of both the colon and the rectum (Figure 8—figure supplement 1E,F), and this phenomenon was invariably associated with CIN in MSS tumors (Figure 8—figure supplement 1G). These findings indicate that focal deletions of Chr 14 with resultant loss of copy number may in part contribute to downregulation of Daple we observe in colorectal cancers.

Next, we asked how Daple expression changes in disseminated tumor cells and serum. Compared to normal subjects, Daple mRNA was elevated in both cell-free RNA samples (Figure 8G) and in tumor cells (Figure 8H) isolated from peripheral circulation of patients with colorectal cancer. We found that expression of Daple in circulating tumor cells (CTCs) of patients with metastatic colorectal cancer was associated with progression of disease/recurrence (Figure 8I) and poor survival (Figure 8J). Furthermore, higher Daple expression in CTCs correlated positively with increased expression of genes that are known to trigger EMT (Figure 8—source data 3). These results indicate that Daple is expressed in disseminated tumor cells and that higher expression is associated with EMT and poorer clinical outcomes.

Taken together, these results define the profile of dysregulated Daple expression during oncogenic progression in the colon (Figure 8K): Daple is first suppressed during adenoma-to-carcinoma progression and expressed later in disseminated tumor cells.

## Discussion

### FZDRs activate Gi proteins via Daple

The major finding in this work is the discovery of a G protein regulatory function in Daple, which activates trimeric G proteins downstream of FZDRs. We provide biochemical and in-cellulo evidence for the presence of a GBA motif that activates Gαi and an independent domain within the C-terminal region of Daple, which directly binds the cytoplasmic tail of FZDRs. Such a coexistence allows Daple to link G protein activation to ligand-activated FZDRs within ternary FZDR-Daple-Gαi complexes at the PM. We also demonstrate that FZDRs and Gαi come within close proximity of each other (∼10 nm based on FRET imaging studies) within these complexes, suggesting a direct interaction between them on the Daple platform. In cells without Daple, or in cells expressing a mutant in which the GBA motif is selectively disrupted, FZDRs and G proteins do not approach each other and G protein is not activated, demonstrating an obligatory role for Daple's GBA motif in the assembly of FZDR-Gαi complexes. These findings provide a new perspective on the role of G proteins in Wnt signaling because previous work has widely debated the fundamental question whether the 7-TM FZDRs can directly bind and activate G proteins. Arguments that have favored the classification of FZDRs as GPCRs are supported by experimental evidence that FZDRs indeed signal via G proteins, for example, structure-based bioinformatic prediction, pertussis toxin sensitive signaling pathways, genetic linkage with G proteins, and ability to bind β-arrestin for subsequent internalization (Slusarski et al., 1997a; Liu et al., 2001, 2005; Ahumada et al., 2002; Katanaev et al., 2005; Gao and Wang, 2006; Ma and Wang, 2006). Arguments that refute such classification highlight the lack of direct experimental proof of G-protein interaction with FZDRs, and that most studies use experimental models (overexpressed receptors or gain-of-function), which do not necessarily implicate necessity (Schulte and Bryja, 2007). Our work breaks the impasse in the field by the discovery of an alternative mechanism of G protein activation by FZDRs: we propose that the C-terminus of Daple is the long sought molecular linker that couples FZDRs to efficient G protein activation by virtue of its ability to simultaneously bind receptors and activate G proteins. However, that some FZDRs may directly couple with other G proteins under certain circumstances cannot be ruled out (see below).

### Daple is a new member in the family of non-receptor GEFs that function via GBA-motif

Here, we demonstrate that Daple is a new member of a family of non-receptor activators of G protein, thereby adding to the growing evidence that trimeric G proteins can be activated by mechanisms differing from classical GPCR-mediated activation. We demonstrated that Daple activates Gi via a signature sequence, that is, the GBA motif that allows proteins and synthetic peptides to exert GEF activity on G proteins and provides a structural basis for non-receptor mediated activation of G proteins (Johnston et al., 2005; Austin et al., 2008; Garcia-Marcos et al., 2009, 2011b). Daple shares overall homology with GIV, the prototype GBA motif-containing protein, and both of them are classified as members of the CCDC88 family. Interestingly, the C-terminal domains of these two proteins, in which their conserved GBA motifs are located, share very little overall similarity. These observations suggest that Daple and GIV arose from a common ancestor protein and that the GBA function was selectively preserved, while the rest of the C-terminal domain diverged in evolution.

Daple has the biochemical features of a GEF: it binds preferentially to inactive, GDP-bound Gαi subunits and accelerates the rate of nucleotide exchange. The GEF activity of Daple, that is, its ability to accelerate the exchange of nucleotide, is more robust than that previously reported for FZDRs in similar in vitro assays (∼2.5–3-fold activation compared to ∼1.5-fold) (Koval and Katanaev, 2011). Inefficient activation of G proteins by FZDRs (∼5–20% efficacy compared to that observed for a ‘classical’ GPCR, i.e., Adenosine 2B receptor) has also been documented in yeast (Nichols et al., 2013), which lack homologues of Daple. Our studies measuring G protein activation in Daple-depleted cells or in cells without a functional GBA motif in Daple help establish an obligatory role of Daple as a *bona fide* G protein activator, which enables FZDRs to indirectly activate Gi to robust levels. These findings cannot rule out other possibilities, for example, that FZDRs may directly activate Gi to a lesser extent under certain circumstances, or that Daple and FZDRs may activate different subsets of G proteins. The latter possibility is exemplified by Gαo, which has been most widely reported as a target for FZDRs (Liu et al., 1999, 2001; Katanaev et al., 2005; Bikkavilli et al., 2008; Katanaev and Buestorf, 2009; Egger-Adam and Katanaev, 2010) but not for Daple (this work). The marked preference of Daple for α-subunits of the Gi family is a common feature shared with previously described GBA proteins; Daple, GIV, Calnuc, NUCB2 (Garcia-Marcos et al., 2009, 2011b), or the synthetic peptides KB-752 and GSP (Johnston et al., 2005; Austin et al., 2008) can exquisitely distinguish between Gαi and Gαo proteins, despite their being closely related and sharing 75% sequence homology.

Although the biochemical properties of Daple as a G protein regulator are similar to those of other proteins with a GBA motif, we provide evidence that the coupling between Daple and Gαi-subunits has unique structural determinants. Daple can bind to two Gαi3 mutants, W258F and K248M, that abolish binding to GIV and Calnuc, respectively. Moreover, we have previously shown (Garcia-Marcos et al., 2010, 2011b) that these mutants are able to discriminate between GIV and Calnuc (K248M binds GIV, but not Calnuc, and W258F binds Calnuc, but not GIV), which further suggest that different GBA-Gαi interactions have unique properties that impart a high degree of specificity. The validated homology models of these GBA-Gαi interactions (Garcia-Marcos et al., 2009, 2011b) offer some clues into the origin of such specificity: despite docking onto the SwII/α3 hydrophobic cleft of Gαi, all GBA-motif containing proteins make additional and unique contacts with Gαi, which generate specificity for each GBA motif. We conclude that the Daple:Gαi interface has unique features that distinguish it from GIV:Gαi or Calnuc:Gαi interfaces, and exploiting such structural specificity may help devise strategies to selectively target the Daple:Gαi interface, and thereby, modulate Wnt signaling.

### G protein regulatory function of Daple is essential for enhancement of non-canonical Wnt signaling

We demonstrate that recruitment of Daple-Gαi complexes to the cytoplasmic tail of ligand activated FZDRs dictates several closely intertwined spatial and temporal aspects of post-receptor signaling events within the non-canonical Wnt pathway. At the immediate post-receptor level, Daple competes with Dvl, the major signaling scaffold for Wnt signaling (Gao and Chen, 2010), for binding to FZDR, and recruits and activates Gαi in close proximity to activated receptors at the PM. That Dvl and Daple/Gαi complexes may compete for binding to FZDR is in keeping with others' findings that overexpression of Dvl interferes with the engagement of Gi proteins with ligand-activated FZDRs (Kilander et al., 2014), and that Dvl is unlikely to directly link G proteins to FZDRs, as proposed by some (Schulte and Bryja, 2007). Once recruited, Daple's GEF activity triggers Gi activation, which leads to inhibition of cellular cAMP via Gαi:GTP and activation of non-canonical Wnt signaling pathways involved in cell motility (e.g., PI3K and Rac1) via ‘free’ Gβγ. The consequences of these signaling mechanism are enhanced formation of actin stress fibers, 2D-cell migration after wounding, 3D-invasion through basement membrane proteins, and upregulation of genes that trigger EMT; all phenotypes that have been previously attributed to enhancement of non-canonical Wnt signaling (Minami et al., 2010). We also show that the FZD7R-Daple-Gi axis suppresses responses associated with tumorigenesis, for example, β-catenin/TCF/LEF signaling, oncogenic transformation, anchorage independent growth, and anchorage-dependent colony formation; all attributable to its ability to activate G proteins via its GBA motif. Because the FZDR-Daple-Gi axis specifically modulates non-canonical Wnt signals, but has no effect on canonical Wnt responses, we conclude that Daple suppresses the canonical β-catenin/TCF/LEF pathway primarily by enhancing the antagonistic non-canonical Wnt pathway (Torres et al., 1996; MacLeod et al., 2007; Ying et al., 2007, 2008; Chien et al., 2009). Although the mechanism(s) by which the non-canonical Wnt pathway inhibits the canonical β-catenin/TCF/LEF pathway remains unclear, and some have proposed that such decisions are made at the level of the receptors (Logan and Nusse, 2004), how Daple-dependent G protein signaling in the vicinity of the receptors may affect this process remains unclear. Our finding that Daple binds preferentially to some FZDRs, and not others, could influence the decision of canonical vs non-canonical Wnt signaling, or alternatively, activation of Gαi and inhibition of cellular cAMP by Daple could directly antagonize a previously described role of the adenylate cyclase/cAMP/PKA pathway in phosphorylating and stabilizing β-catenin (Hino et al., 2005). Regardless of the mechanism(s) involved, activation of Gi and enhancement of non-canonical Wnt signaling are accompanied by the suppression of the canonical β-catenin pathway in cells expressing Daple-WT, which correlates with all the key anti-growth and anti-transformation phenotypes that define a tumor suppressor/anti-oncogene (Cooper, 2000). Although it is possible that some of the effects of the FZDR-Daple-Gi axis in tumor suppression are mediated by the destabilization of β-catenin, further investigations are required to clarify this point. We conclude that the G protein regulatory function of Daple is essential for enhancing at least two major cellular phenotypes previously attributed to non-canonical Wnt signaling (McDonald and Silver, 2009), suppression of cell transformation and growth, and enhancement of cell invasion. As for potential implications in other key cellular processes that are deregulated during cancer progression, it is noteworthy that non-canonical Wnt signaling has also been demonstrated to play a crucial role in planar cell polarity and asymmetric cell division in stem cells (Bentzinger et al., 2014, 2013). Several studies have shown that the Wnt7A/FZD7 pathway establishes front-rear cell polarity and directional migration of human myogenic progenitors and facilitate the extension of satellite stem cells, all by activating PI3K/Akt pathway and Rac1 (Bentzinger et al., 2014, 2013). Because one of the major roles of the FZDR-Daple-Gi axis is enhancement of PI3K and Rac1 activities, it is possible that this axis also aids in the establishment of cell polarity and/or the maintenance of stem-ness via enhancement of the non-canonical Wnt pathway. Further studies are required to determine if such is the case.

### Daple expression and non-canonical Wnt signaling are similarly dysregulated during oncogenesis

We showed here that Daple is downregulated during oncogenesis at the step of conversion from adenoma to carcinoma, and that lower expression of Daple in the primary tumor is associated with higher frequency of cancer recurrence. We describe that expression of Daple in CTCs correlated with an increased EMT signature, disease progression (growth of current metastasis or formation of new metastasis), and poorer survival. This bimodal dysregulation (suppressed first, expressed later) and bi-faceted role (tumor suppressor in the normal epithelium, but enhancer of tumor invasion in cancer cells) during cancer progression mirrors what was previously unequivocally documented for the non-canonical Wnt5a signaling (McDonald and Silver, 2009)—Wnt5a signaling is suppressed earlier to allow cellular transformation and tumor growth, and enhanced later during tumor invasion. However, molecular mechanisms for such bimodal deregulation of the bi-faceted non-canonical Wnt pathway remain poorly understood. Such phenomenon is not restricted to Daple or the Wnt pathway, because major signaling programs like the TGFβ-SMAD pathway have also been shown to display similar bimodal deregulation and a bi-faceted role (Akhurst and Derynck, 2001), and a similar phenomenon is observed in the case of Daple's closely related orthologue, GIV (Ghosh et al., 2010): downregulation of GIV by alternative splicing triggered proliferation early during tumor growth, whereas an increase in GIV by transcriptional upregulation enhanced cell invasion later during oncogenesis. Because Daple serves as a *bona fide* enhancer of the non-canonical Wnt pathway, we conclude that upregulation or downregulation in Daple expression contributes, at least in part, to the bimodal deregulation of the Wnt5a signaling pathway observed in cancers.

We also demonstrate that the mechanism for downregulation of Daple in cancers follows typical tumor suppressor genetics during neoplastic transformation (Payne and Kemp, 2005). Downregulation of Daple mRNA coincided with adenoma-to-carcinoma transition, and the frequency of such downregulation in the primary tumor directly correlated with the degree of CIN. A loss of copy number of Daple DNA, and consequent downregulation of gene expression and function was noted in the primary tumors, predominantly among the tumors with CIN. This pattern is in keeping with the well-documented role of CIN in generating loss of heterozygosity (LOH) and haploinsufficiency of other tumor suppressors (Sotillo et al., 2009). In the case of Daple, such insufficiency is likely to increase the fitness of cells that have undergone such a LOH because depletion of Daple suppresses non-canonical Wnt signaling and allows unrestricted propagation of canonical Wnt pathways. Consequently, proliferation/growth is triggered, which enables these cells to rapidly outcompete the remaining population. Based on the location of ccdc88c (Daple gene) at a site on the long arm of Chr 14, which is known to be frequently deleted in a variety of cancers (Suzuki et al., 1989; Hu et al., 2002; Rouault et al., 2012), we conclude that tumors harboring a focal deletion at that site are in part driven by insufficient expression of the tumor suppressor Daple. Additional mechanisms, for example, alternative splicing may further contribute to oncogenesis via dysregulation of Daple expression, as described in a rare and fatal human developmental anomaly (Ekici et al., 2010). This anomaly was attributed to deregulation of Wnt signaling due to a loss of Daple's 29th exon, which contains the G protein regulatory GBA motif. Although many other mechanisms may be involved, loss of Daple expression, or a selective loss of its G protein regulatory function has emerged as a final common pathway, which disrupts Daple-Gαi axis of Wnt signaling and derails tissue homeostasis.

The precise molecular mechanism(s) that enhances Daple expression or function and consequently triggers an EMT signature and cell invasion during cancer progression remains unclear. Transcriptional compensation for loss of an allele (Guidi et al., 2004) or gain-of-function mutations (van Oijen and Slootweg, 2000) is possible mechanisms, as shown previously in the case of other tumors suppressors. In this regard, it is noteworthy that although Daple bound the cytoplasmic tails of several FZDRs to varying extent, the preference for FZD7R was striking and may provide some clues as to why/how Daple may enhance tumor progression. Although all FZDRs promiscuously interact with more than one of the many Wnt isoforms to activate canonical and/or non-canonical Wnt signaling (King et al., 2012), FZD7 stands out as a receptor that functions at the cross-roads of canonical and non-canonical Wnt signaling pathways in a unique way. FZD7R is a downstream target of β-catenin in cancer cells (Barker and Clevers, 2006), and consequently, enhanced canonical Wnt signaling upregulates FZD7R expression during cancer progression. It has been proposed that such increased FZD7R expression due to aberrant canonical Wnt signals may serve as a positive forward-feedback mechanism to perpetuate Wnt/β-catenin signaling, thus, facilitating colorectal cancer progression and metastasis. Because Daple appears to be upregulated during cancer invasion and in circulating cancer cells (and such upregulation is associated with worse prognosis) and enhances non-canonical Wnt signaling downstream of FZD7R, it is possible that Daple's functional interaction with this receptor further enhances prometastatic signaling via amplification of the non-canonical Wnt pathway, which synergizes with the previously proposed forward-feedback canonical Wnt signaling loop during cancer progression. We conclude that such preferential signaling downstream of FZD7R and the temporal profile of expression of Daple are well-poised to suppress or enhance non-canonical Wnt signaling and aid in different steps of tumor progression (see legend for Figure 8L).

In conclusion, we have defined Daple as a novel regulator of G protein activity, which directly binds FZDRs and enables these 7-TM receptors to recruit and activate Gi, and trigger non-canonical Wnt signaling to suppress tumorigenesis and enhance tumor invasion. These findings set a new paradigm for the long-debated mechanisms by which FZDRs are coupled to G protein activation. As a potent tumor suppressor with multiple intriguing domains, for example, the newly identified GBA and the Frizzled-binding domain, Daple presents many signaling interfaces that could be developed as targets for modulating Wnt signaling. Because its levels of expression in primary tumors, circulating cell-free transcripts and in CTCs may indicate tumor characteristics, Daple presents many avenues for further development as clinically useful diagnostic and prognostic biomarkers.

## Materials and methods

### Reagents and antibodies

Unless otherwise indicated, all reagents were of analytical grade and obtained from Sigma–Aldrich (St. Louis, MO). Cell culture media were purchased from Invitrogen. All restriction endonucleases and *Escherichia coli* strain DH5α were purchased from New England Biolabs (Ipswich, MA). *E. coli* strain BL21 (DE3), phalloidin-Texas Red were purchased from Invitrogen (Grand Island, NY). Genejuice transfection reagent was from Novagen (Madison, WI). PfuUltra DNA polymerase was purchased from Stratagene (La Jolla, CA). Recombinant Wnt3a and Wnt5a were purified as previously described (Willert, 2008). Briefly, conditioned media (CM) were collected the day after confluence was reached. WNT proteins were purified from 6 liters of CHO CM. CM was complemented with 1% Triton X-100 (vol/vol), 20 mM Tris-Cl pH 7.5, and 0.01% NaN_3_. Goat anti-rabbit and goat anti-mouse Alexa Fluor 680 or IRDye 800 F(ab′)2 used for immunoblotting (IB) were from Li-Cor Biosciences (Lincoln, NE). Mouse anti-His, anti-FLAG (M2), anti-α tubulin, and anti-actin were obtained from Sigma; anti-Myc and anti-HA were obtained from Cell Signaling Technology (Beverly, MA) and Covance (Princeton, NJ), respectively. Rabbit anti-pan-Gβ (M-14), anti-Gαi3, anti-DVL, and anti-β-catenin were obtained from Santa Cruz Biotechnology (Dallas, TX); anti-Akt and phospho-Akt (S473) were obtained from Cell Signaling; anti-Rac1 was obtained from BD Transduction Laboratories (San Jose, CA). Anti-Daple antibodies were generated in collaboration with Millipore (Carlsbad, CA) using the C-terminus of Daple (aa 1660–2028) as an immunogen.

### Plasmid constructs and mutagenesis

Cloning of N-terminally tagged myc-Daple was carried out in two steps by piece-meal assembly. A fragment of hDaple obtained from Kazusa (KIAA1509; clone fh14721, inserted into pBluescript II SK [+]) was used as a source of 3′ nucleotide bp 2131–6087. The N-terminus of hDaple was artificially synthesized (Genscript, San Diego) and used as a source for the 5′ nucleotide bp 1–2130. The full-length hDaple gene (corresponding to the Ref Seq NM_001080414.3 [mRNA] and NP_001073883.2 [protein]) was assembled by inserting 5′ and 3′ fragments into pcDNA 3.1 between *NotI/EcoRI* and *EcoRI/BamHI*, respectively. The *EcoRI* cloning site in the middle of the Daple sequence was eliminated by mutagenesis. The entire gene length was sequenced prior to cloning it into myc-pcDNA 3.1 (+) between *KpnI/EcoRI* to generate myc-Daple. All subsequent site-directed mutagenesis and truncated constructs (myc-Daple full-length F1675A (FA), myc-Daple deleted from aa 2025–2028 (ΔPBM), myc-Daple FA+ΔPBM (2M), and myc-Daple CT 1650–2028 aa) were carried out on this template using Quick Change as per manufacturer's protocol. The GST-Daple-CT WT, His-Daple-CT WT, and FA constructs (1650–1880 aa and 1650–2028 aa) used for in vitro protein–protein interaction assays were cloned from myc-Daple pcDNA 3.1 and inserted within the pGEX-4T or pET28b vectors, respectively, between *NdeI*/*EcoRI* restriction sites.

The HA-tagged FZD7R construct was generated by cloning the human receptor (ATCC# 10658884; Gen Bank BC015915.1; Ref Seq: NM_003507.1) in pcDNA 3.1 between *HindIII/EcoRI* and by subsequently inserting a HA tag at the C-terminus by mutagenesis. FZD7R-CFP construct was a generous gift from Carl-Philip Heisenberg (Institute of Science and Technology, Austria) (Witzel et al., 2006). Gαi3-YFP and Gαi1-YFP (internally tagged Gαi subunits: the coding sequence for YFP was inserted in the αb–αc loop after Ala-121 of Giα1 and Ala-114 of Giα3, which does not affect their biochemical properties), CFP-Gβ_1_ and untagged Gγ are a generous gift from Moritz Bunemann (Philipps-Universität Marburg, Germany) (Bunemann et al., 2003; Gibson and Gilman, 2006). Mouse Dvl1 and HA-Ras G12V were generous gifts from Mikhail V Semenov (Harvard Medical School) and Robert Hayward (London, UK), respectively.

Cloning of rat Gα-proteins into pGEX-4T-1 (GST-Gαi3, GST-Gαi1, GST-Gαi2, and GST-Gαo), GST-Gαi3 K248M and W258F; His-Gαi3; Gαi3-FLAG; Gαi3-HA; and GST-GIV CT 1671–1755 aa has been described previously (Ghosh et al., 2008; Garcia-Marcos et al., 2009, 2010, 2011b; Ghosh et al., 2010). GST-tagged C-termini of FZDRs 3–7 (Yao et al., 2004) were generous gifts from Ryoji Yao (JFCR research institute, Japan). The C-terminal cytoplasmic tails of human FZD1 (aa 614–647) and mouse FZD2 (aa 537–570) were cloned into the BamHI/ EcoRI sites of pGEX-4T-1 to generate the plasmids for bacterial expression of GST-FZD1-CT and GST-FZD2-CT, respectively. GST-PBD was a generous gift from Gary Bokoch (The Scripps Research Institute, La Jolla).

Daple shRNA constructs were created using the following approach. Promising targets at the 3′ UTR region of human Daple (NM_001080414) were identified using the pSicoOligomaker software. The two most promising hits were chosen based on favorable score (>7). Duplexed oligos were designed against those targets and cloned in pSico Puro vector between HpaI and XhoI. Details of targets for hDaple sequence and oligos used are provided below:

Targets for hDAPLE 3′ UTR (coding DNA sequence is from bp 155–6241)

6570 GTAGAACACTCATTTGCAA (shRNA 1)

6929 GCACCTGCCTTCCTAGATT (shRNA 2)

hDaple sh1 forward 5′ TGTAGAACACTCATTTGCAATTCAAGAGATTGCAAATGAGTGTTCTACTTTTTTC

hDaple sh1 reverse 5′ TCGAGAAAAAAGTAGAACACTCATTTGCAATCTCTTGAATTGCAAATGAGTGTTCTACA

hDaple sh2 forward 5′ TGCACCTGCCTTCCTAGATTTTCAAGAGAAATCTAGGAAGGCAGGTGCTTTTTTC

hDaple sh2 reverse 5′ TCGAGAAAAAAGCACCTGCCTTCCTAGATTTCTCTTGAAAATCTAGGAAGGCAGGTGCA

### Protein expression and purification

GST and His-tagged recombinant proteins were expressed in *E. coli* strain BL21 (DE3) (Invitrogen) and purified as described previously (Ghosh et al., 2008, 2010; Garcia-Marcos et al., 2011a). Briefly, bacterial cultures were induced overnight at 25°C with 1 mM isopropylβ-D-1-thio-galactopyranoside (IPTG). Pelleted bacteria from 1 l of culture were resuspended in 20 ml GST-lysis buffer (25 mM Tris·HCl, pH 7.5, 20 mM NaCl, 1 mM Ethylenediaminetetraacetic acid (EDTA), 20% [vol/vol] glycerol, 1% [vol/vol] Triton X-100, 2 × protease inhibitor mixture [Complete EDTA-free; Roche Diagnostics]) or in 20 ml His-lysis buffer (50 mM NaH_2_PO_4_ [pH 7.4], 300 mM NaCl, 10 mM imidazole, 1% [vol/vol] Triton X-100, 2 × protease inhibitor mixture [Complete EDTA-free; Roche Diagnostics]) for GST or His-fused proteins, respectively. After sonication (three cycles, with pulses lasting 30 s/cycle, and with 2 min interval between cycles to prevent heating), lysates were centrifuged at 12,000×*g* at 4°C for 20 min. Except for GST-FZD and GST-PBD constructs (see in vitro GST pulldown assay section), solubilized proteins were affinity purified on glutathione-Sepharose 4B beads (GE Healthcare) or HisPur Cobalt Resin (Pierce), dialyzed overnight against PBS, and stored at −80°C.

### Cell culture and the rationale for choice of cells in various assays

Tissue culture was carried out essentially as described before (Ghosh et al., 2008, 2010; Garcia-Marcos et al., 2011a). We used a total of five different cell lines in this work, each chosen carefully based on its level of endogenous Daple expression and the type of assay. All these cell lines were cultured according to ATCC guidelines.

Cos7 cells were primarily used for transient overexpression of tagged Daple or Dvl proteins and lysates of these cells were used as source of proteins in various protein–protein interaction (IP and pulldown) assays. We chose to carry out these assays in Cos7 cells because they are easily and efficiently transfected (>90% efficiency) with most constructs. The added advantage is that they have no detectable endogenous Daple (by IB and qPCR) and provide a system to selectively analyze the properties of WT vs mutant Daple constructs without interference from endogenous Daple.

HeLa cells were primarily used to study the in-cellulo dynamics of interaction between Daple and FZD7R during non-canonical Wnt signaling because those cells have been extensively used to study Wnt5a-stimulated non-canonical signaling by various groups (Yamamoto et al., 2007; Sato et al., 2010). We noted that HeLa cells have low amounts of endogenous Daple, and that it was an adequate system to study the role of Daple in cells because Wnt5a stimulation could trigger the previously described downstream signaling responses in our hands (Yamamoto et al., 2007; Sato et al., 2010). Noteworthy, the efficiency of transient transfection of various Daple constructs in these cells was >90%, as determined by immunofluorescence staining.

HEK293T cells were used exclusively for FRET and co-IP studies involving FZD7R/G proteins because these cells are widely used and preferred for such studies involving GPCR/G protein signaling due to several reasons. HEK293 cells are the single most widely used cell line for heterologous expression (both transient and stable expression) of GPCRs (Thomas and Smart, 2005) because they allow a robust expression of functional receptors compared to most cells (Massotte, 2003; Thomas and Smart, 2005). Microarray analyses have confirmed that they have an adequate transcriptome that supports various elements of GPCR/G protein signaling pathways, for example, GPCR ligands, trimeric G proteins, scaffolding components that mediate receptor endocytosis, kinases, and phosphatases that phosphoregulate GPCR functions, and so on (Atwood et al., 2011). We have confirmed that they express endogenous Daple as a full-length protein, at physiologic levels, and the localization of Daple (as determined by immunofluorescence) is primarily at the PM (data not shown), where FZDRs are activated.

Low passage NIH3T3 fibroblasts were used exclusively in 3-D Matrigel invasion assays and in neoplastic transformation assays to study the role of Daple in suppressing growth in soft agar upon Ras-mediated transformation. The rationale for their use in invasion assay lies in the fact that non-transformed NIH3T3 fibroblasts are poorly invasive in vitro and non-tumorigenic and non-metastatic in animal studies (Bondy et al., 1985; Hill et al., 1988; Chambers et al., 1990; Tuck et al., 1991). It is because of this reason, NIH3T3 cells are widely used to study proteins that can trigger a gain in invasive properties (Leitner et al., 2011). For the neoplastic transformation assays, we used Ras-transformed NIH3T3 cells because this is the gold standard assay used to study the role of a gene/protein in tumor transformation (Egan et al., 1987). The rationale for using NIH3T3 in both the above assays is further strengthened by the fact that they are highly transfectable (∼80% transfection efficiency with myc-Daple) and express Daple at very low-endogenous levels (as determined by IB and qPCR) compared to normal colonic epithelium. Such expression pattern allows us to study the effect of various mutant Daple constructs without significant interference due to the endogenous protein.

DLD1 were primarily used to study the effect of Daple on cancer cell growth properties (anchorage-dependent and independent) and to assess the effect of Daple on the classical Wnt signaling pathway (β-catenin/TCF/LEF). There are several reasons why this cell line was chosen: (1) We focused on colorectal cancer in this study, and DLD1 cells were appropriate to translate our findings because they are human colorectal cancer cells; (2) We determined that levels of Daple are significantly lower (∼10-fold) in these cells compared to normal colon (data not shown), thereby allowing us to study the effect of various mutant Daple constructs without significant interference due to the endogenous protein; (3) These cells have been extensively characterized with respect to most oncogenes (ATCC database) and are highly tumorigenic in 2-D and 3-D cultures due to a mutation in KRAS (G13D) (Shirasawa et al., 1993; Ahmed et al., 2013); (4) They are a sensitive model to study how various manipulations of the non-canonical Wnt signaling pathway oppose the canonical Wnt pathway during tumor growth because they constitutively secrete Wnt ligands to maintain high levels of the canonical signaling (Voloshanenko et al., 2013) within the growth matrix. Production and secretion of endogenous ligands bypasses the need to add exogenous ligands repeatedly during prolonged assays that last ∼2 weeks.

### Transfection, generation of stable cell lines and cell lysis

Transfection was carried out using Genejuice (Novagen) for DNA plasmids following the manufacturers' protocols. HeLa and DLD1 cell lines stably expressing Daple constructs were selected after transfection in the presence of 800 µg/ml G418 for 6 weeks. The resultant multiclonal pool was subsequently maintained in the presence of 500 µg/ml G418. Daple expression was verified independently using anti-Myc and anti-Daple antibodies by IB and estimated to be ∼5× the endogenous level. Unless otherwise indicated, for assays involving serum starvation, serum concentration was reduced to 0.2% FBS overnight for HeLa cells and 0% FBS for Cos7, HEK293T, and DLD1 cells.

Whole-cell lysates were prepared after washing cells with cold PBS prior to resuspending and boiling them in sample buffer. Lysates used as a source of proteins in IP or pull-down assays were prepared by resuspending cells in Tx-100 lysis buffer (20 mM HEPES[4-(2-hydroxyethyl)-1-piperazineethanesulfonic acid], pH 7.2, 5 mM Mg-acetate, 125 mM K-acetate, 0.4% Triton X-100, 1 mM Dithiothreitol (DTT), supplemented with sodium orthovanadate [500 µM], phosphatase [Sigma], and protease [Roche] inhibitor cocktails), after which they were passed through a 28G needle at 4°C, and cleared (10,000×*g* for 10 min) before use in subsequent experiments.

### Quantitative Immunoblotting (IB)

For immunoblotting, protein samples were separated by sodium dodecyl sulfate polyacrylamide gel electrophoresis (SDS-PAGE) and transferred to polyvinylidene difluoride (PVDF) membranes (Millipore). Membranes were blocked with phosphate buffer saline (PBS) supplemented with 5% non-fat milk (or with 5% bovine serum albumin (BSA) when probing for phosphorylated proteins) before incubation with primary antibodies. Infrared imaging with two-color detection and band densitometry quantifications were performed using a Li-Cor Odyssey imaging system exactly as done previously (Garcia-Marcos et al., 2010, 2011a, 2011b, 2012; Ghosh et al., 2010). All Odyssey images were processed using ImageJ software (NIH) and assembled into figure panels using Photoshop and Illustrator software (Adobe).

### In vitro GST pulldown and IP assays

Purified GST-Gαi3 or GST alone (5 µg) was immobilized on glutathione-Sepharose beads and incubated with binding buffer (50 mM Tris-HCl [pH 7.4], 100 mM NaCl, 0.4% [vol:vol] Nonidet P-40, 10 mM MgCl_2_, 5 mM EDTA, 30 µM GDP, 2 mM DTT, protease inhibitor mixture) for 90 min at room temperature as described before (Ghosh et al., 2008, 2010; Lin et al., 2011; Garcia-Marcos et al., 2011a). Lysates (∼250 µg) of Cos7 cells expressing appropriate myc-Daple constructs or purified His-Daple-CT (aa 1650–2028) protein (3 µg) were added to each tube, and binding reactions were carried out for 4 hr at 4°C with constant tumbling in binding buffer (50 mM Tris-HCl [pH 7.4], 100 mM NaCl, 0.4% [vol:vol] Nonidet P-40, 10 mM MgCl_2_, 5 mM EDTA, 30 µM GDP, 2 mM DTT). Beads were washed (4×) with 1 ml of wash buffer (4.3 mM Na_2_HPO_4_, 1.4 mM KH_2_PO_4_ [pH 7.4], 137 mM NaCl, 2.7 mM KCl, 0.1% [vol:vol] Tween 20, 10 mM MgCl_2_, 5 mM EDTA, 30 µM GDP, 2 mM DTT) and boiled in Laemmli's sample buffer. In some experiments, the ‘active’ conformation of the G protein was stabilized by replacing GDP in the binding and wash buffers with 30 µM GTPγS or a mixture of 30 µM GDP/30 µM AlCl_3_/10 mM NaF. Immunoblot quantification was performed by infrared imaging following the manufacturer's protocols using an Odyssey imaging system (Li-Cor Biosciences).

GST-FZD7-CT and GST-PBD constructs were immobilized on glutathione-Sepharose beads directly from bacterial lysates by overnight incubation at 4°C with constant tumbling. Next morning, GST-FZD7-CT immobilized on glutathione beads were washed and subsequently incubated with His-tagged Daple-CT or Gαi3 proteins at 4°C with constant tumbling. Washes and IB were performed as previously.

For IP, cell lysates (∼1–2 mg of protein) were incubated for 4 hr at 4°C with 2 μg of appropriate antibody, anti-HA mAb (Covance) for HA-Gαi3 or HA-FZD7, anti-FLAG (M2 from Sigma) mAb for FLAG-Gαi3, or their respective pre-immune control IgGs. Protein G (for all mAbs) Sepharose beads (GE Healthcare) were added and incubated at 4°C for an additional 60 min. Beads were washed in PBS-T buffer (4.3 mM Na_2_HPO_4_, 1.4 mM KH_2_PO_4_, pH 7.4, 137 mM NaCl, 2.7 mM KCl, 0.1% [vol:vol] Tween 20, 10 mM MgCl_2_, 5 mM EDTA, 2 mM DTT, 0.5 mM sodium orthovanadate), and bound proteins were eluted by boiling in Laemmli's sample buffer.

### Homology modeling

The structure of the synthetic peptide KB-752 bound to Gαi1 (PDB:1Y3A) was used as the template to generate the modeling project in Deep View/Swiss-PdbViewer v3.7 for Daple (aa 1668–1679) in complex with Gαi3. The modeling project was submitted to the Swiss-Model Server (http://swissmodel.expasy.org//SWISS-MODEL.html) (Schwede et al., 2003), and model images were generated by MolsoftICM (San Diego, CA).

### Steady-state GTPase assays

Under the experimental conditions of steady-state GTPase assays, GTP hydrolysis occurs as a two-step reaction, that is, (1) GDP is released from the G protein and exchanged for GTP and (2) the GTP loaded is hydrolyzed. Nucleotide exchange is the rate limiting step in this process because it is ∼50–100 times slower than GTP hydrolysis by Gαi subunits (Mukhopadhyay and Ross, 2002). Thus, the steady-state GTPase activity reflects the rate of nucleotide exchange and was performed as described previously (Garcia-Marcos et al., 2010, 2011b, 2012). Briefly, His-Gαi3 (100 nM) was preincubated with different concentrations of His-Daple-CT (aa 1650–2028) for 15 min at 30°C in assay buffer (20 mM Na-HEPES, pH 8, 100 mM NaCl, 1 mM EDTA, 2 mM MgCl_2_, 1 mM DTT, 0.05% [wt:vol] C12E10). GTPase reactions were initiated at 30°C by adding an equal volume of assay buffer containing 1 µM [γ-^32^P]GTP (∼50 c.p.m/fmol). For the time course experiments, duplicate aliquots (50 μl) were removed at different time points and reactions stopped with 950 μl ice-cold 5% (wt/vol) activated charcoal in 20 mM H_3_PO_4_, pH 3. For the dose–dependence curve experiments, reactions were stopped at 15 min. Samples were then centrifuged for 10 min at 10,000×*g*, and 500 μl of the resultant supernatant was scintillation counted to quantify released [^32^P]P_i_. For the time course experiments, data were expressed as raw c.p.m. For the dose–dependence curve experiments, the background [^32^P]P_i_ detected at 15 min in the absence of G protein was subtracted from each reaction and data expressed as percentage of the P_i_ produced by His-Gαi3 in the absence of His-Daple-CT.

### GTPγS-binding assays

GTPγS binding was measured using a filter binding method as described previously (Garcia-Marcos et al., 2010, 2011b). His-Gαi3 (100 nM) was preincubated with different concentrations of His-Daple-CT (aa 1650–2028) for 15 min at 30°C in assay buffer (20 mM Na-HEPES, pH 8, 100 mM NaCl, 1 mM EDTA, 25 mM MgCl_2_, 1 mM DTT, 0.05% [wt:vol] C12E10). Reactions were initiated at 30°C by adding an equal volume of assay buffer containing 1 µM [^35^S] GTPγS (∼50 c.p.m/fmol). Duplicate aliquots (25 μl) were removed at different time points, and binding of radioactive nucleotide was stopped by addition of 3 ml ice-cold wash buffer (20 mm Tris-HCl, pH 8.0, 100 mm NaCl, 25 mm MgCl_2_). The quenched reactions were rapidly passed through BA-85 nitrocellulose filters (GE Healthcare) and washed with 4 ml wash buffer. Filters were dried and subjected to liquid scintillation counting. To determine the specific nucleotide binding, the background [^35^S] GTPγS detected in the absence of G protein was subtracted from each reaction and data expressed as percentage of the [^35^S] GTPγS bound by His-Gαi3 in the absence of His-Daple-CT.

### FRET studies

FRET experiments were performed using the classical ECFP- and EYFP-tagged proteins as donor and acceptor FRET-probe pairs, respectively. Previously validated and published FZD7-CFP construct was a generous gift from Carl-Philip Heisenberg (Witzel et al., 2006). Previously validated Gαi3-YFP and Gαi1-YFP (internally tagged Gαi subunits) and CFP-Gβ_1_ were generous gifts from Moritz Bunemann (Bunemann et al., 2003; Gibson and Gilman, 2006). Interaction of FZD7-CFP and Gαi3-YFP proteins was studied in HEK293T cells using a Leica inverted laser scanning confocal microscope. Axial scans of 0.5 µ thickness that resolved most of the PM from a single cell were chosen for imaging and the signal in the donor and acceptor channels was ensured to be in mesoscopic regime to avoid inhomogeneity's between samples (Midde et al., 2014). Loss of FRET upon Gi activation and heterotrimer dissociation was measured between Gαi1-YFP and CFP-Gβ_1_ proteins co-expressed in living HeLa cells using Olympus FV1000 inverted confocal laser scanning microscope equipped with a 60× 1.49 N.A oil immersed objective designed to minimize chromatic aberration and enhance resolution for 405–605 nm imaging as described previously (Midde et al., 2015). Images were sequentially acquired through Donor, FRET, and acceptor channels using 405 and 488 laser lines to excite CFP and YFP, respectively. FRET efficiency was calculated on a pixel by pixel basis from ratiometric images obtained in individual channels (donor, acceptor, and FRET) through a RiFRET plugin in ImageJ software (Roszik et al., 2009). All images are corrected for the spectral cross-talk obtained from cells transfected with either donor or acceptor probes alone. Regions of interest were randomly drawn at the PM (an example is shown in Figure 2—figure supplement 1E; red circle) to compute FRET efficiency.

### Gαi activity as determined by anti-Gαi:GTP mAb

For IP of active Gαi3, freshly prepared cell lysates (2–4 mg) were incubated for 30 min at 4°C with the conformational Gαi:GTP mouse antibody (1 μg) (Lane et al., 2008b) or with control mouse IgG. Protein G Sepharose beads (GE Healthcare) were added and incubated at 4°C for additional 30 min (total duration of assay is 1 hr). Beads were immediately washed three times using 1 ml of lysis buffer (composition exactly as above; no nucleotides added), and immune complexes were eluted by boiling in SDS as previously described (Lopez-Sanchez et al., 2014).

### Measurement of cAMP

HeLa cells were transfected with Daple-WT or Daple-FA, serum starved (0.2% FBS, 16 hr) and incubated with isobutylmethylxanthine (IBMX, 200 µM, 20 min) followed by Wnt5A stimulation (100 ng/ml, 20 min) and Forskolin (10 μM, 10 min). To stop the reaction, cell medium was replaced with 150 μl of ice-cold TCA 7.5% (wt/vol). cAMP content in TCA extracts was determined by radioimmunoassay and normalized to the amount of protein (determined using a dyebinding protein assay [Bio-Rad]) per sample as previously described (Ostrom et al., 2001).

### Gβγ displacement assays

This assay was performed as described previously (Garcia-Marcos et al., 2009). Briefly, GST alone or GST-Gαi3 proteins immobilized on glutathione-agarose beads were incubated overnight at 4°C with HEK293T cell lysates in binding buffer (50 mM Tris-HCl, pH 7.4, 100 mM NaCl, 0.4% [vol:vol] NP-40, 10 mM MgCl_2_, 5 mM EDTA, 2 mM DTT, protease inhibitor cocktail supplemented with 30 µM GDP). Unbound Gβγ-subunits were washed twice with the same buffer and proteins bound to the glutathione-agarose beads divided into equal aliquots containing ∼5 µg (∼0.4 µM) GST-fusion proteins. Aliquots were incubated with increasing concentrations (0.05–1 µM) of purified His-Daple-CT (1650–2028) wild-type or 1 µM His-Daple-CT F1675A in binding buffer supplemented with GDP (∼200 µl) for 5 hr at 4°C. Glutathione-agarose beads were washed and bound proteins eluted by boiling in Laemmli sample buffer and separated by SDS-PAGE.

### Rac1 activity assays

Rac1 activity in HeLa cells lines was monitored using GST-tagged PAK1-binding domain (PBD; pGEX-PBD) as described previously (Benard and Bokoch, 2002). Briefly, *E. coli* strain BL21 bacteria transformed with pGEX-PBD were grown at 37°C, and GST-PBD expression was induced at OD600 with 1 mM IPTG for 3 hr at 37°C with shaking. Bacterial lysates were prepared as described above in protein purification section, cleared of debris by centrifugation and subsequently aliquots of lysates were stored at −80°C until use. Aliquots of bacterial lysates were thawed, cleared of precipitated proteins by centrifugation at 14,000×*g* for 20 min, and the cleared supernatant was subsequently incubated with glutathione beads overnight at 4°C with constant tumbling to prepare purified bead-bound GST-PBD freshly for each assay.

To analyze the role of Daple in regulation of Rac1 activity, we used HeLa cells. For assays done on cells at steady-state, cells were maintained overnight in a media containing 2% or 0.2% FBS prior to lysis. Lysis was carried out first in RIPA buffer (20 mM HEPES pH 7.4, 180 mM NaCl, 1 mM EDTA, 1% Triton X-100, 0.5% sodium deoxycholate, 0.1% SDS, supplemented with 1mMDTT, sodium orthovanadate [500 μM], phosphatase [Sigma], and protease [Roche] inhibitor mixtures) for 15 min on ice, and then for an additional 15 min after addition of an equal volume of Triton X-100 lysis buffer (20 mM Hepes [pH 7.2], 5 mM Mg-acetate, 125 mM K-acetate, 0.4% Triton X-100, 1 mM DTT, supplemented with sodium orthovanadate [500 μM], phosphatase [Sigma], and protease [Roche] inhibitor mixtures). During the second 15 min of incubation, cells were broken by passing through a 28-gage needle at 4°C and lysates were subsequently cleared (10,000×*g* for 10 min) before use. For assays done with/without ligand stimulation, HeLa cells serum-starved (0.2% FBS) overnight and subsequently treated or not with 100 ng/ml Wnt5a for 5 min at prior lysis as above. Equal aliquots of lysates were incubated with bead-bound GST-PBD for 1 hr at 4°C with constant tumbling. Beads were washed in PBS-T buffer (4.3 mM Na_2_HPO_4_, 1.4 mM KH_2_PO_4_, pH 7.4, 137 mM NaCl, 2.7 mM KCl, 0.1% [vol:vol] Tween 20, 10 mM MgCl_2_, 5 mM EDTA, 2 mM DTT, 0.5 mM sodium orthovanadate) and bound proteins were eluted by boiling in Laemmli's sample buffer.

### Immunofluorescence

HeLa cell lines were fixed at room temperature with 3% paraformaldehyde for 20–25 min, permeabilized (0.2% Triton X-100) for 45 min, and incubated for 1 hr each with primary and then secondary antibodies as described previously (Ghosh et al., 2008). Dilutions of antibodies and reagents were as follows: Myc (1:500); Phalloidin (1:1000); DAPI (1:2000); goat anti-mouse (488 and 594) Alexa-conjugated antibodies (1:500); anti-phospho-Histone H3 (Ser28) (1:150). Cells were imaged on a Leica SPE confocal microscope using a 63× oil objective and 488, 561, and 405 laser lines for excitation (Lopez-Sanchez et al., 2014). All individual images were processed using ImageJ software and assembled for presentation using Photoshop and Illustrator software (Adobe).

### β-catenin reporter assays

These assays were carried out using the well-established reporter 7xTcf-eGFP (7TGP) (Fuerer and Nusse, 2010). Stable cells lines expressing this reporter were generated by lentiviral transduction and subsequent selection using standard procedures. Lentiviral infection and selection were performed according to standard procedures. Briefly, 10-cm plates DLD1 cells at 70% confluency were incubated with media containing 8 µg/ml polybrene and 10 µl of lentivirus for 6 hr. After 24 hr post-infection, selection of puromycin-resistant clones was initiated by adding the antibiotic at 2 µg/ml final concentration. The resultant DLD1-7TGP stable cells were subsequently transfected with various myc-Daple constructs and selected for G418 resistance as described earlier in methods. The DLD1-7TGP cells stably expressing myc-Daple were incubated overnight at 0.2% FBS, analyzed by fluorescence microscopy, and photographed prior to lysis. Whole-cell lysates samples were then boiled in Laemmli's sample buffer, and GFP protein expression was monitored by IB.

### Scratch-wounding, trans-well chemotaxis, and 3D-matrigel invasion assays

Scratch-wound assays were done as described previously (Ghosh et al., 2008). Briefly, monolayer cultures (100% confluent) of HeLa cells expressing Daple WT or Daple FA were scratch-wounded using a 20-μl pipette tip and incubated in 2% FBS media. The cells were subsequently monitored by phase-contrast microscopy over the next 24 hr. To quantify cell migration (expressed as percent of wound closure), images were analyzed using ImageJ software to calculate the difference between the wound area at 0 hr and that at 12 hr divided by the area at 0 hr × 100.

Chemotactic cell migration assays were performed using Corning Transwell plates according to the manufacturer's protocol. HeLa cells were trypsinized, counted, and placed in a Transwell with media containing 0.2% FBS (75000 cells/well). Media in the bottom chamber of each well were supplemented with 0.2% FBS and 100 ng/ml Wnt5a to trigger chemotactic migration. Cells were allowed to migrate for 24 hr and fixed prior staining. Cells that had successfully migrated to the side of the permeable membrane facing the bottom chamber were visualized by staining the membrane with Giemsa. Cell migration (expressed as number of cells/high-power field) was quantified by analyzing 15–20 random fields per membrane insert per condition for the number of Giemsa stained cells.

NIH3T3 cell invasion assay in 3D culture was performed according to the manufacturer's protocol (Trevigen, Cultrex 3D Spheroid BME Cell Invasion Assay, catalog # 3500-096-K). Briefly, non-invasive NIH3T3 cells (∼3000 cells) transfected with empty vector (control) or myc-Daple constructs were incubated first in the Spheroid Formation extracellular matrix containing 0.2% FBS for 3 days. Invasion matrix was then added and layered on top with media containing FBS. Serum-triggered cell invasion was photographed under light microscope everyday for 10 days, and fresh media (FBS concentration is increased each time in order to maintain a gradient) were replenished every 48 hr. Photographs were analyzed and pseudocolored by ImageJ to reflect cell density.

### Analysis of mitotic index

The mitosis rate of HeLa cells stably expressing Daple-WT and Daple-FA was measured by phospho-Histone H3 (Ser28) (mitotic index) exactly as we did previously (Ghosh et al., 2010). Mitotic index was determined by dividing the number of positively stained cells/the total number of DAPI-stained nuclei × 100.

### Transformation assay

Neoplastic transformationin Ras-transformed NIH3T3 fibroblasts were analyzed using standard assays of colony formation in soft agar as described previously (Clark et al., 1995). Low-passage NIH3T3 cells (∼5000) stably co-transfected with appropriate myc-Daple construct (2 µg cDNA) and HA-Ras G12V (1 µg cDNA) were analyzed for their ability to form tumor foci in soft agar plates. Plates were incubated in 5% CO_2_ at 37°C for ∼2 weeks in growth media supplemented with 2% FBS. They were finally incubated with 0.1% (wt/vol) 3-(4,5-dimethylthiazol-2-yl)2 2,5-diphenyl tetrazolium bromide (MTT; Sigma) in PBS for 1 hr to visualize colonies. The remaining NIH3T3 cells not used for this assay were lysed and analyzed for myc-Daple and Ha-Ras G12V expression by IB.

### Anchorage-independent tumor growth assay

Anchorage-independent growth of DLD1 cells was analyzed in agar as described previously (Provost et al., 2012). Briefly, petri plates (60 mm) were pre-layered with 3 ml 1% Bacto agar (Life Technologies) in Dulbecco's Modified Eagle's medium (DMEM) containing 10% Fetal Bovine Serum (FBS). Approximately ∼5000 DLD1 cells stably expressing various Daple constructs were then plated on top in 3 ml of 0.3% agar–DMEM with 10% FBS. All assays were carried out using three replicate plates at a seeding density of ∼5000 cells/plate. Following overnight incubation in 5% CO_2_ incubator, 1 ml DMEM supplemented with 2% FBS was added to maintain hydration. After 2 weeks of growth, colonies were stained with 0.005% crystal violet/methanol for 1 hr and subsequently photographed by light microscopy. The number of colonies in ∼15–20 randomly-selected fields was counted under 10× magnification. The remaining DLD1 cells were lysed and analyzed by IB to confirm Daple construct expression. Each experiment was analyzed in triplicate.

### Anchorage-dependent tumor growth assay

Anchorage-dependent growth was monitored on solid (plastic) surface as described previously (Franken et al., 2006). Briefly, anchorage-dependent growth was monitored on solid (plastic) surface. Approximately ∼1000 DLD1 cells stably expressing various Daple constructs were plated in 6-well plates and incubated in 5% CO_2_ at 37°C for ∼2 weeks in 0.2% FBS growth media. Colonies were then stained with 0.005% crystal violet for 1 hr. The remaining DLD1 cells were lysed and analyzed by IB to confirm Daple construct expression. Each experiment was analyzed in triplicate.

### Ccdc88c (DAPLE) mRNA analysis in CTCs from patients with metastatic colorectal carcinoma

51 patients with metastatic colorectal cancer from the Complexo Hospitalario Universitario de Santiago de Compostela, Spain were enrolled (Barbazán et al., 2012). All participants signed an informed consent specifically approved for this study by the Ethical Committee of the Complexo Hospitalario Universitario of Santiago de Compostela (code of approval: 2009/289). Inclusion criteria were the presence of measurable metastatic colorectal cancer (stage IV) and an Eastern Cooperative Oncology Group (ECOG) performance status not greater than 2. Disease progression, evaluated by computerized tomography, was defined following RECIST 1.1 guidelines (1) as an increase in the number of metastatic lesions, growth of existing lesions in more than 20% or both during treatment. Furthermore, 24 healthy individuals with similar age ranges to those of patients were included as negative controls.

CTCs were isolated using an EpCAM-based immunoisolation (dynabeads) using the CELLection Epithelial Enrich kit (Life Technologies), and CTC RNA was purified with the Qiamp Viral kit (Qiagen) as previously described (Barbazán et al., 2012). Briefly, Superscript III based cDNA synthesis (Life Technologies) was carried out to preamplify a region within the coiled-coil domain of Daple to maximize posterior detection rates (TaqMan Preamp kit, Applied Biosystems). Preamplified samples were subsequently subjected to TaqMan real-time PCR amplification (Applied Biosystems) (probe numbers Hs00380245_m1 and Hs00325884_m1). Non-specific blood cells in the CTC-enriched isolates were accounted for by analyzing the expression of CD45 as a lymphoid cell marker (not present in cancer cells). All the results for Daple are normalized with the expression of CD45 (in all sample types). Briefly, the Ct value (coming from qPCRs) for Daple and CD45 are subtracted to 40 (maximum number of cycles in qPCR) to get an intuitive value (more value, more expression). Daple 40-ct values are normalized with those from CD45, afterwards.

### RNA isolation and qPCR

Total RNA was isolated using an RNeasy kit (QIAGEN) as per the manufacturers' protocol. First-strand cDNA was synthesized using Superscript II reverse transcriptase (Invitrogen), followed by ribonuclease H treatment (Invitrogen) prior to performing quantitative real-time PCR. Reactions omitting reverse transcriptase were performed in each experiment as negative controls. Reactions were then run on a real-time PCR system (ABI StepOnePlus; Applied Biosystems). Gene expression was detected with SYBR green (Invitrogen), and relative gene expression was determined by normalizing to GAPDH using the ΔC_T_ method.

Primer sequences are listed as follows:

**Table:** 

Gene	Forward	Reverse
Daple-CC	5′-TGA CAT GGA GAC CCT GAA GGC TGA-3′	5′-TTTCATGCGGGCCTCACTGCTGA-3′
GAPDH	5′-TCA GTT GTA GGC AAG CTG CGA CGT-3′	5′-AAGCCAGAGGCTGGTACCTAGAAC-3′
LOXL3	5′-ATGGGTGCTATCCACCTGAG-3′	5′-GAGTCGGATCCTGGTCTCTG-3′
Vim	5′-AAGAGAACTTTGCCGTTGAA-3′	5′-GTGATGCTGAGAAGTTTCGT-3′
SFPR-1	5′-GAGTTTGCACTGAGGATGAAAA-3′	5′-GCTTCTTCTTCTTGGGGACA-3′
AXIN-2	5′-GAGTGGACTTGTGCCGACTTCA-3′	5′-GGTGGCTGGTGCAAAGACATAG-3′
OPN	5′-TTGCAGCCTTCTCAGCCAA-3′	5′-GGAGGCAAAAGCAAATCACTG-3′

### Analysis of Daple mRNA expression in advanced adenomas and cancers

Advanced adenomas were collected and analyzed as described previously (Toiyama et al., 2013). All patients provided written informed consent and the study was approved by institutional review boards of Baylor University Medical Center, Dallas, USA and the Okayama University Hospital, Okayama, Japan. Colorectal carcinomas used in this work were derived from a previously well-characterized, chemo-naive, stage II colorectal cancer cohort from Munich (Nitsche et al., 2012). The ethics committee of the Klinikum rechts der Isar, Munich, Germany, approved collection of the patient samples (#1926/07, and #5428/12). All samples were obtained after prior informed written consent. For each sample, 20 to 30 mg of frozen tumor tissue was removed for further analysis using a cryostat microtome (CM3050 S, Leica Microsystems, Wetzlar, Germany). Histology-guided sample selection (Maak et al., 2013) was performed by a pathologist to ensure a sufficient amount of tumor cells (good cellularity and >30% tumor cells). RNA was obtained using the Qiagen AllPrep DNA/RNA Mini Kit (Qiagen GmbH, Hilden, Germany) according to the manufacturer's protocol. Subsequently qPCR was performed as described above.

### Data analysis and statistics

All experiments were repeated at least three times, and results were presented either as one representative experiment or as average ±SD or SEM. Statistical significance was assessed with two-tailed Student's *t*-test.

Statistical evaluation for CTC studies were performed using IBM SPSS Statistics Version 19 (SPSS Inc., IBM Corporation, Somers, New York, USA). In order to derive optimal cut-off values of Daple expression levels, maximally selected log-rank statistics performed by R Software version 2.13.0 (R Foundation for Statistical Computing, Vienna, Austria) were used. To consider multiple test issue within these analyses, the R-function *maxstat.test* was employed (Hothorn and Zeileis, 2008). Time-dependent survival probabilities were estimated with the Kaplan–Meier method, and the log-rank test was used to compare independent subgroups. All statistical tests were performed two-sided, and p-values less than 0.05 were considered to be statistically significant.
