# Supplementary material for: Daple is a novel non-receptor GEF required for trimeric G protein activation in Wnt signaling
Source: eLife. 2015 Jun 30;4:e07091. doi: 10.7554/eLife.07091 (PMC4484057; doi:10.7554/eLife.07091)
Supplement: Figure 8—source data 1. — The publicly available GSE database, a system to store, retrieve, and analyze all types of high-throughput microarray data was used to compare the levels of expression of Daple mRNA in colorectal cancer vs matched normal controls. From left to right, the columns indicate the GSE series ID, the PMID number for the respective source manuscripts, total samples analyzed in each study, fold change in Daple mRNA observed, and the significance (p-value) of any changes observed. A meta-analysis combining the p-values from these studies was analyzed by Fisher's method and displayed as bar graphs in Figure 8A. DOI: http://dx.doi.org/10.7554/eLife.07091.019 [file elife07091s001.doc]

**Figure 8-source data 1 :**

**Meta-Analysis of Daple mRNA Expression in Colorectal Cancer vs Matched Normal Colon**

| **GSE Series ID** | **Reference** | **Total Samples Analyzed (Cancer/Normal)** | **Fold Change**  **(mRNA)** | **p-Value** |
| --- | --- | --- | --- | --- |
| GSE21815- Gene expression profiles in laser microdissected colorectal cancer tissues | PMID: 21862635 | 132/8 | ↓2.01 | 0.0108 |
| GSE21510- Clinical Significance of Osteoprotegrin Expression in Human Colorectal Cancer | PMID: 21270110 | 148 | ↓1.9 | 8.8E-5 |
| GSE22598- Clinical Significance of UNC5B Expression in Colorectal Cancer | PMID: 21922135 | 17 (paired cancer and normal) | ↓1.69 | 0.0027 |
| GSE18105 - Colorectal cancer gene expression profile | PMID: 20162577 | 77 (paired cancer and normal) | ↓1.68 | 0.0031 |
| GSE41328 - Colon adenocacinoma and matched normal tissue - Lab 2 | PMID: 17160039 | 5 ( paired cancer and normal) | ↓1.63 | 0.0139 |
| GSE41328 - Colon adenocacinoma and matched normal tissue - Lab 2 (different primer pair) | PMID: 17160039 | 5 ( paired cancer and normal) | ↓1.58 | 0.0087 |
| GSE5350_GPL570 - Microarray Quality Control (MAQC) Project | PMID: 16964229 | 10 ( paired cancer and normal) | ↓1.52 | 0.0001 |
