# Supplementary material for: Daple is a novel non-receptor GEF required for trimeric G protein activation in Wnt signaling
Source: eLife. 2015 Jun 30;4:e07091. doi: 10.7554/eLife.07091 (PMC4484057; doi:10.7554/eLife.07091)
Supplement: Figure 8—source data 2. — The publicly available GSE database was used to compare the levels of expression of Daple mRNA in MSI vs MSS colorectal cancers. From left to right, the columns indicate the GSE series ID, the PMID number for the respective source manuscripts, total samples analyzed in each study, fold change in Daple mRNA observed, and the significance (p-value) of any changes observed. A meta-analysis combining the p-values from these studies was analyzed by Fisher's method and displayed as bar graphs in Figure 8C. DOI: http://dx.doi.org/10.7554/eLife.07091.020 [file elife07091s002.doc]

**Figure 8-source data 2:**

**Meta-Analysis of Daple mRNA Expression in Microsatellite Unstable (MSI) vs Stable (MSS) Colorectal Cancers**

| **GSE Series ID** | **Reference** | **Total Samples Analyzed (Cancer/Normal)** | **Fold Change**  **(mRNA)** | **p-Value** |
| --- | --- | --- | --- | --- |
| GSE**13294- Expression data from primary colorectal cancers** | PMID: 19088021 | 155 | ↓1.49 | 2.9 E-8 |
| GSE**4554- Gene expression signature of colorectal cancer with microsatellite instability** | PMID: 17047040 | 84 | ↓2.11 | 6.5 E-5 |
| **GSE2138- Colon cancer profiling** | PMID: 16247484 | 20 | ↓1.78 | 0.0181 |
| **GSE13067 - Expression data from primary colorectal cancers** | PMID: 19088021 | 74 | ↓1.31 | 0.0415 |
