# Supplementary material for: Daple is a novel non-receptor GEF required for trimeric G protein activation in Wnt signaling
Source: eLife. 2015 Jun 30;4:e07091. doi: 10.7554/eLife.07091 (PMC4484057; doi:10.7554/eLife.07091)
Supplement: Figure 8—source data 3. — Expression of Daple, ZEB2, and LOXL3 mRNA were analyzed in CTCs immunoisolated from 50 patients with metastatic colorectal cancer. An analysis of the Pearson's correlation coefficient for each pair of genes shows that higher expression of Daple is significantly associated with higher expression of ZEB2 and LOXL3, two genes implicated in triggering EMT. DOI: http://dx.doi.org/10.7554/eLife.07091.021 [file elife07091s003.doc]

**Figure 8-source data 3:**

**Daple expression in CTCs positively correlates with markers of EMT**

|  |  | **CCDC88c (Daple)** | **ZEB2** | **LOXL3** |
| --- | --- | --- | --- | --- |
| **CCDC88c (Daple)** | Pearson Correlation  P value (2-tailed)  N (# patients) | 1  50 | 0.894  0.000  50 | 0.776  0.000  50 |
| **ZEB2** | Pearson Correlation  P value (2-tailed)  N (# patients) | 0.894  0.000  50 | 1  50 | 0.797  0.000  50 |
| **LOXL3** | Pearson Correlation  P value (2-tailed)  N (# patients) | 0.776  0.000  50 | 0.797  0.000  50 | 1  50 |
